# Accuracy of Answers to Cell Lineage Questions Depends on Single-Cell Genomics Data Quality and Quantity

**DOI:** 10.1371/journal.pcbi.1004983

**Published:** 2016-06-13

**Authors:** Adam Spiro, Ehud Shapiro

**Affiliations:** Department of Computer Science and Applied Mathematics and Department of Biological Chemistry, Weizmann Institute of Science, Rehovot, Israel; University of Zurich, SWITZERLAND

## Abstract

Advances in single-cell (SC) genomics enable commensurate improvements in methods for uncovering lineage relations among individual cells, as determined by phylogenetic analysis of the somatic mutations harbored by each cell. Theoretically, complete and accurate knowledge of the genome of each cell of an individual can produce an extremely accurate cell lineage tree of that individual. However, the reality of SC genomics is that such complete and accurate knowledge would be wanting, in quality and in quantity, for the foreseeable future. In this paper we offer a framework for systematically exploring the feasibility of answering cell lineage questions based on SC somatic mutational analysis, as a function of SC genomics data quality and quantity. We take into consideration the current limitations of SC genomics in terms of mutation data quality, most notably amplification bias and allele dropouts (ADO), as well as cost, which puts practical limits on mutation data quantity obtained from each cell as well as on cell sample density. We do so by generating *in silico* cell lineage trees using a dedicated formal language, eSTG, and show how the ability to answer correctly a cell lineage question depends on the quality and quantity of the SC mutation data. The presented framework can serve as a baseline for the potential of current SC genomics to unravel cell lineage dynamics, as well as the potential contributions of future advancement, both biochemical and computational, for the task.

## Author Summary

A human cell lineage tree describes the entire developmental dynamics of a person starting from the zygote and ending with each and every extant cell. Fundamental open problems in biology and medicine are in fact questions about the human cell lineage tree: its structure and its dynamics in development, growth, renewal, aging, and disease. Consequently, a method to know the human cell lineage tree would allow resolving these problems and enable a leapfrog advance in human knowledge and health. Recent advancements in single-cell genomics have the potential to uncover various properties of the human cell lineage tree and thus promote our understanding of various biological phenomena. In this paper we present a computational framework along with specific results, which enable to understand what can be achieved using the limitations of current technologies and predict future capabilities based on future improvements. This approach can serve as a valuable tool for researchers who plan to perform lineage experiments both in designing and optimizing the actual experimental needs and predicting the costs and limitations of the plan. This work can also help researchers focus on developing what is needed for future advancements.

## Introduction

Recent advances in SC technologies have generated a unique opportunity to delineate the complex behavior of heterogeneous cell populations and uncover their underlying mechanistic dynamics [1]. The use of SC genomics to reveal cell lineage relationships have been recently demonstrated in various scenarios including diseases such as cancer [2–6] and normal development [7–10]. Lineage analysis of cells sampled from an organism makes use of somatic mutations to discover common history dynamics of the sampled cells. There are several types of somatic mutations that can be used for this task, including Single Nucleotide Variations (SNV) [2, 3, 11–13], Short Tandem Repeats (STR, also called Microsatellites) [6, 8–10, 14–18], Copy Number Variations (CNV) [4, 5, 7], and Transposable Elements (TE) [8] where each type has a different mutational model and different mutation rates. This analysis is mostly effective when analyzing SC since the mixed mutational signal of cell bulks does not allow delineating mutational co-occurrences and cannot distinguish between subpopulations with different mutational patterns. Although published work have shown the great potential of using SC mutational analysis for unraveling cell lineage dynamics, there are still several major limitations, which hamper further generalization of this concept to various biological questions and prevent its use in large scale experiments. These limitations include 1) technical issues related to SC genomics, including the need for DNA amplification that introduces technical noise, 2) lack of high throughput SC isolation techniques, especially if one wants to retain the original 3D structure, or analyze rare cell types that are difficult to isolate, 3) associated costs, such as Whole Genome Amplification (WGA) kits, sequencing costs, and other consumable products (e.g., reagents and microfluidic devices), and 4) lack of computational infrastructure and dedicated algorithms specifically designed for the unique challenges of SC genomics.

The feasibility of using somatic mutations for uncovering cell lineage dynamics is dependent on these issues but also on the specifics of the pursued biological question. Some factors are inherent, such as the mutation rate and number of cell divisions, but others can be overcome by spending more money or by improving biochemical or computational procedures. Using controlled *ex-vivo* experiments is a close approximation to real biological scenarios; however, it can be very costly and laborious. Furthermore, many scenarios cannot be examined due to technical limitations in trying to mimic real biological dynamics (e.g., cell differentiation leading to changes in cellular dynamics), and also various parameter combinations cannot be studied using an *ex-vivo* experiment. A computational alternative is to model and simulate various biological scenarios using a range of parameters and conditions. Not only this approach enables to inspect the strengths and weaknesses of existing methods, it can also enable to predict the impact of future improvements.

Until now, there has not been any systematic examination of how much mutational data is required in order to accurately answer questions related to the structure and dynamics of SC lineage trees. In this work we cover few common biological settings, which capture certain tree properties such as depth (corresponds to number of cell divisions) and clustering relationships, in order to systematically evaluate the feasibility of answering cell lineage questions using somatic mutations, and predict future capabilities by extending the range of parameters values to represent future enhancements. Using mutational data from an *ex-vivo* experiment we estimated and modeled the properties of the mutational signal quality, afflicted mainly by the random noise and ADO caused during the preprocessing and amplification of SC DNA. We then applied this model onto the signal of simulated lineage trees, generated using a dedicated formal language and simulation tool, based on environment-dependent Stochastic Tree Grammars (eSTG) [19], which is capable of generating both the entire modeled cell lineage tree and the corresponding somatic mutations accumulated through cell divisions. We present the results on a variety of parameters values, including different distance relationships (corresponding to different number of cell divisions) between different cell types, different mutation rates and two types of somatic mutations, including STR [20] and SNV. We also take into consideration current estimated costs of biochemical analysis and for each combination of parameters we calculate the cost-optimized number of cell samples and genomic loci that enable to answer the biological question with high confidence. We map the dependency between the quality and quantity of the SC mutational data and the ability to answer cell lineage questions of specific settings, which can be used as a framework for planning cell lineage experiments and predicting the potential of future enhancements, both biochemical and computational.

## Results

We have previously presented a formal language, called eSTG, for describing population dynamics [19] and a corresponding programming and simulation environment, called eSTGt (eSTG tool) [21]. The language captures in broad terms the effect of the changing environment while abstracting away details on interaction among individuals. A prominent feature of the tool is that it can stochastically produce lineage trees, each corresponding to a different stochastic program execution. These lineage trees record the entire execution history of the process, including the dynamics that led to existing as well as to extinct individuals. In this paper we simulated cell lineage trees using eSTGt by specifying and executing eSTG programs. The output of each program’s execution is an instance of a stochastic lineage tree, which also includes the corresponding somatic mutations as specified by the eSTG programs. By running multiple executions of the programs we collected sufficient statistics as described below. The program specifications used in this manuscript can be found in S1 File.

As mentioned above there are several types of endogenous somatic mutations, including STR, SNV, CNV and TE. Since CNV and TE have a complex dynamics and are hard to predict and model we decided to focus on STR and SNV, which are the most appropriate candidates for inferring general cell lineages retrospectively. For STR mutations, we used the stepwise mutation model [22], which assigns an equal probability *p*_*STR*_ for either an increase or a decrease of one repeat unit during each cell division (see Methods). Current estimations of the STR mutation rate *p*_*STR*_ range between 10^−3^–10^−5^ mutations per locus per cell division depending on various factors such as the STR length, repeat type and the specific cell genotype [20]. The low mutation rate might correspond to short STRs of normal cells whereas the fast mutation rate might correspond to cells harboring Microsatellite Instability, which is common in various types of cancer cells [23]. In order to cover the entire spectrum we chose to simulate three scales of mutation rates, namely, *p*_*STR*_ = 10^−3^, 10^−4^, 10^−5^. SNV mutations were modeled by randomly mutating each base with probability *p*_*SNV*_ following each cell division. The mutation rate *p*_*SNV*_ is estimated to be between 10^−7^–10^−10^ mutations per nucleotide per cell division [24]. Since mutation rate of 10^−10^ was too low to yield any significant signal we present results only for mutation rates *p*_*SNV*_ = 10^−7^, 10^−8^, 10^−9^.

As we mentioned, SC genomics poses many challenges, since the starting material consists of only one copy of each DNA molecule. DNA isolation and amplification introduce technical noise and methods for measuring and reducing it, both biochemically and computationally, are still under extensive research [1]. We chose to model two types of interferences, namely, ADO and random noise. To this end, we used data from an *ex-vivo* experiment that consisted of clonal expansions from which SCs were sampled and processed. The processing included SC Whole Genome Amplification (WGA) and sequencing of targeted loci. ADO was modeled by taking into consideration both the distribution of samples quality and genomic location, and noise was estimated by comparing the genotype of duplicates, which should be identical (see Methods). After simulating the lineage trees along with their somatic mutations we applied the models of the ADO and noise in order to generate the final mutation table that was used for further analysis. In addition, we also adjusted the parameters of the ADO and noise models in order to predict the performance of future improvements in the processing of SC genomics (see Methods). In the figures below we present results for STR using mutation rate *p*_*STR*_ = 10^−4^, which may correspond to highly mutable long STR loci of normal cells, for both current and future predicted signals. Results for the other STR mutation rates (10^−3^, 10^−5^) and for SNV (with mutation rates 10^−7^, 10^−8^, 10^−9^) for current and future signal quality are presented in the Supplementary Information.

In order to optimize the cost efficiency of a specific analysis, we used a fixed ratio of 1:1000 between the analysis cost of a single cell and the analysis cost of a single STR locus, thus one can tradeoff between the number of cells and the number of loci analyzed, depending on specific constraints such as sample scarcity or sequencing availability. In the examples below we used fixed costs of 10$ for a single cell analysis and 0.01$ for a single STR locus. These costs are based on rough estimations of current processing (e.g., WGA kits and consumables) and sequencing costs (see Methods) and can of course be adjusted as needed.

### Reconstruction of Triplet Subtrees

**Fig 1:**
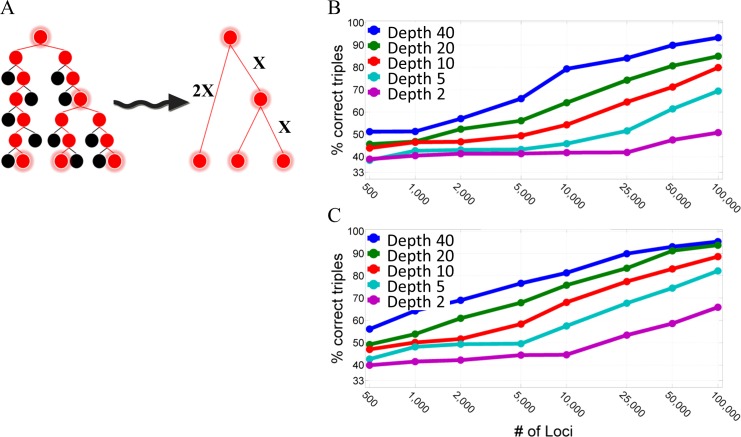
Reconstruction of triplet trees. **(A)** A triplet tree with the indicated depths (X = number of cell divisions) between the root and the leaves and between the root and the branch. **(B)** Reconstruction accuracy as a function of the number of STR loci and the depth of the leaves (number of cell divisions from the root). Results are shown for STR mutation rate of 10^−4^ mutations per locus per cell division. The graph shows that fewer mutations are needed when there are more cell divisions. The results are averaged over 1000 repeated stochastic simulations. **(C)** Same as (B) but using parameters that represent future enhancements (see Methods).

A triplet tree consists of three leaves sampled from a (full) tree and the subtree they induce on the full tree (Fig 1A). Since there are three possible bifurcation arrangements for the triplet tree, the probability of a random triplet tree reconstruction to correctly reconstruct its topology is 1/3. In order to measure the ability to correctly reconstruct a triplet tree using somatic mutations we simulated such trees with various number of cell divisions along with the corresponding mutational signal, which was distorted with the calibrated ADO and noise. We then measured the percentage of correct reconstructions over 1000 repeated stochastic simulations. Fig 1B shows the percentage of correctly reconstructed triplet trees with various number of cell divisions (X = 2,5,10,20,40, see Fig 1A) as a function of the number of analyzed loci (ranging from 500 to 100,000) using STR mutations with mutation rate 10^−4^. Fig 1C shows the results that correspond to future signal improvements. It can be seen, for example, that using 5 cell divisions (X = 5) and 25,000 loci the probability of correctly reconstructing a triplet tree is about 50% (compared to 33% for random reconstruction) using the current signal and almost 70% using the predicted future enhancements. Results for the other STR mutation rates and SNV are presented in S2 File.

### Identifying Depth Differences

Many lineage questions are in fact questions about the depth relationship between two cell groups. Examples include questions related both to cancer dynamics and normal development or renewal. For example, is relapse after chemotherapy caused by ordinary tumor cells escaping chemotherapy stochastically, or by a separate population of rarely-dividing cancer-initiating cells that escape chemotherapy due to their slow division rate [6]? If relapse is initiated from slowly dividing cells, these cells would accumulate fewer mutations since they go through fewer cell divisions. By measuring the distance of the cells from the root of the tree (which can be estimated using a combination of unrelated cell bulks) we can compare the depth relationship between different cell groups. Another example question is whether the adult oocyte pool can be renewed during adulthood [10]? Again, by comparing the number of cell divisions between young and adult female, we may know whether oocytes are generated postnatally.

**Fig 2:**
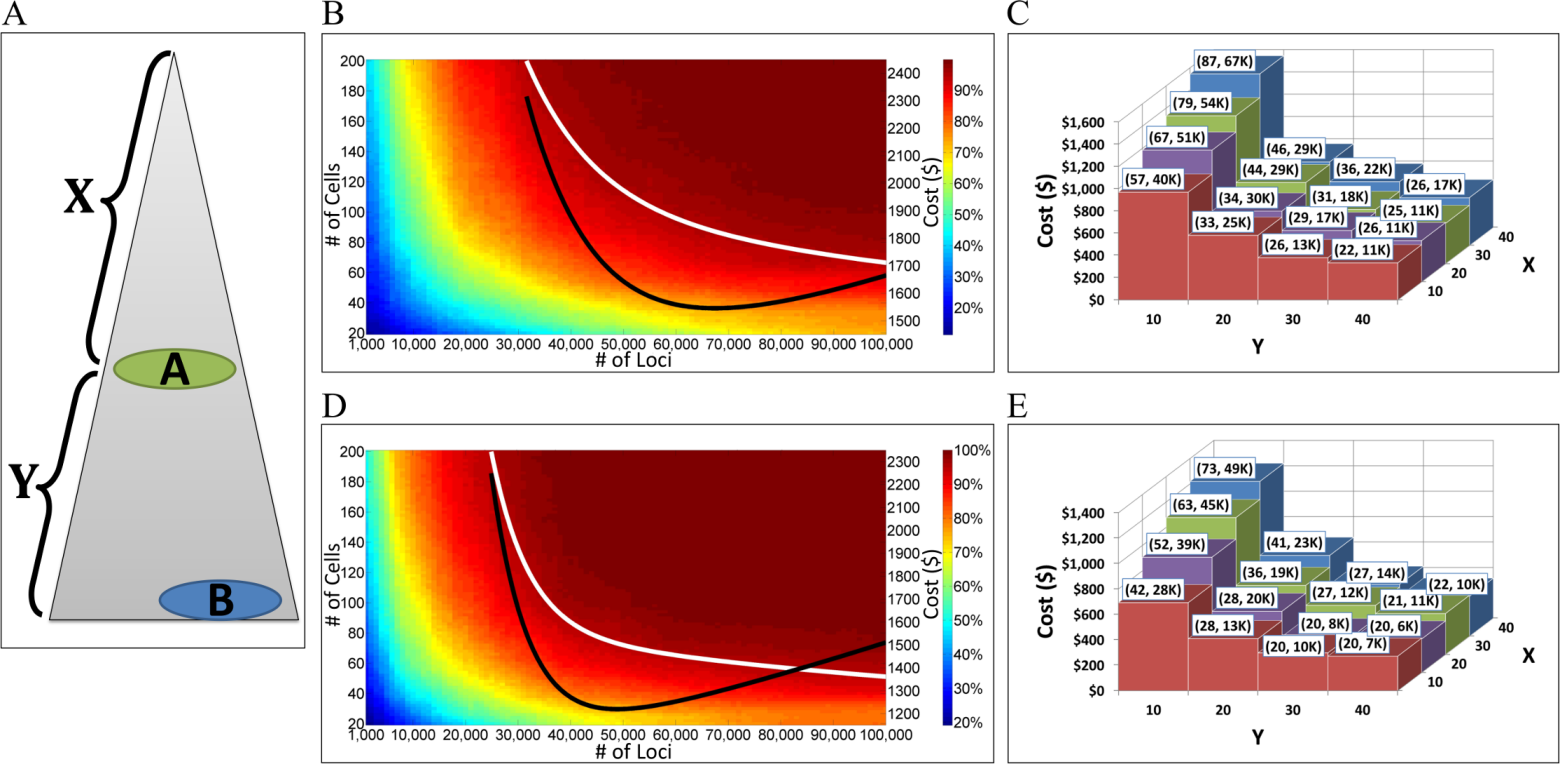
Experiment requirements for identifying that two cell groups have different depth on the cell lineage tree. **(A)** Cells from two cell groups are sampled from the cell lineage tree. The depth (number of cell divisions since the root) of cells of group A is X and the depth of cells of group B is X+Y. **(B)** The heatmap colors represent the statistical power, i.e., the probability of detecting a depth difference between cells from A and cells from B when such difference does exists, as a function of the number of cells (x-axis) and number of loci (left y-axis) analyzed. The probability of falsely identifying depth difference when it does not exist (type I error) is 5% (see Methods). White line marks the area of power = 95%. Black line indicates the overall analysis cost as shown in the right y-axis–both lines have the same x-axis and every point in the black line represents the cost that corresponds to the combination of the number of loci and number of cells as represented by the white line. In this case, for X = 40 and Y = 10, a minimum cost is obtained using about 65K loci and 90 cells. The same power can be obtained using about 35K loci and 200 cells but the cost increases by about 50%. Results are averaged over 1000 stochastic simulations using STR mutation rate of 10^−4^. **(C)** Cost optimization for the number of loci and number of cell samples needed for statistical power of 95%, for various values of X and Y. Numbers in parenthesis indicate the number of cell samples and number of required loci respectively. **(D)** Same as (B) but using parameters that represent future enhancements affecting both the quantity and the quality of the signal (see Methods). **(E)** Same as (C) but using parameters that represent future enhancements.

In order to map the feasibility of answering such questions we simulated lineage trees and analyzed two cell groups from different depths in the tree (Fig 2A). For each cell we estimated its relative depth in the tree using its mutational signature and performed a statistical test that compared the relative depth of cells from both groups (see Methods). Fig 2B shows a heatmap that represents the probability of correctly identifying a significant depth difference between the two cell groups, one of depth X and the other of depth X+Y, for X = 40 and Y = 10, as a function of the number of analyzed cells and the number of analyzed genomic loci. It can be seen that in order to obtain a specific success probability one can tradeoff between the number of analyzed samples and the number of analyzed loci (white line in Fig 2B that represents success probability of 95%), however, a minimum cost can be obtained by selecting the combination that corresponds to the minimum of the black line in Fig 2B that shows the corresponding analysis cost. Fig 2C shows a summary of the cost-optimized number of samples and number of loci needed for obtaining success probability of 95% using various combinations of X and Y corresponding to various depths of the two cell groups (as depicted in Fig 2A). Fig 2D and 2E show the performance using enhanced parameters that correspond to future enhancements in SC genomics. Results for the other STR mutation rates and SNV are presented in S2 File.

### Identifying Independent Subclones

Identifying the clonal relationship between two cell populations arises in many contexts. For example, do progenitor cells commit to a single cell-type or can they produce multiple types as needed [25]? Does geographic separation imply lineage separation or do cells migrate from one area to another [8]? Are the original tumor and its relapse independent clones [6]? The mutational signature of two cell populations can be used to perform clustering analysis in order to examine whether they are separated or intermixed in the lineage tree.

**Fig 3:**
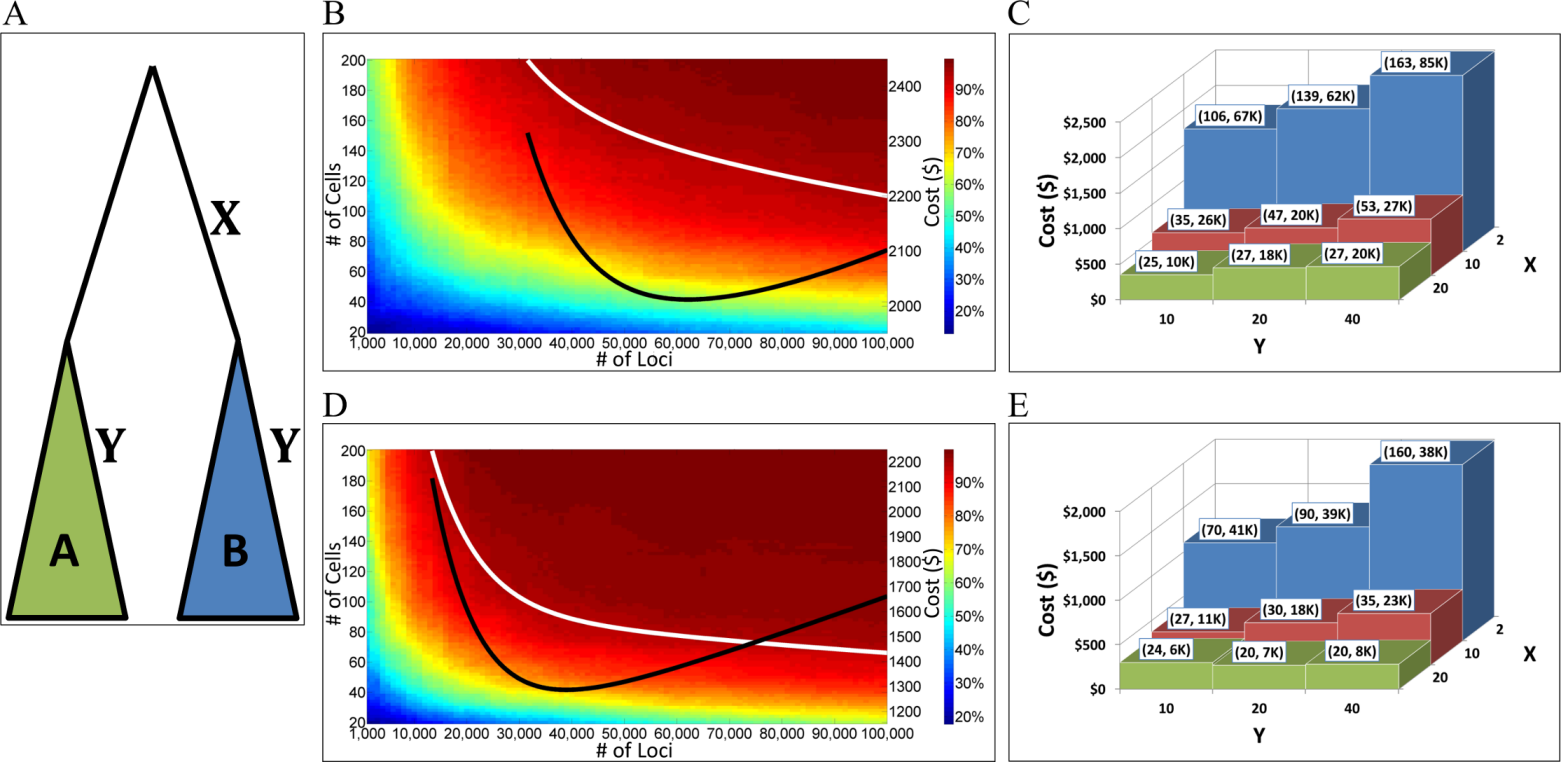
Experiment requirements for identifying that two cell groups are independent subclones. **(A)** The common root of clones A and B divides X times and two random cells of depth X generate the two clones, which divide Y times such that the depth of the extant cells of A and B is X+Y. **(B)** The heatmap colors represent the statistical power, i.e., the probability of correctly identifying the two clones as independent, as a function of the number of cells (x-axis) and number of loci (left y-axis) analyzed. The probability of falsely identifying that the two clones are separated when in fact they are mixed (type I error) is 5% (see Methods). White line marks the area of power = 95%. Black line indicates the overall analysis cost as shown in the right y-axis–both lines have the same x-axis and every point in the black line represents the cost that corresponds to the combination of the number of loci and number of cells as represented by the white line. In this case, for X = 2 and Y = 20, a minimum cost is obtained using about 60K loci and 140 cells. The same power can be obtained using about 30K loci and 200 cells but the cost increases by about 30%. Results are averaged over 1000 stochastic simulations using STR mutation rate of 10^−4^. **(C)** Cost optimization for the number of loci and number of cell samples needed for statistical power of 95%, for various values of X and Y. Numbers in parenthesis indicate the number of cell samples and number of required loci respectively. **(D)** Same as (B) but using parameters that represent future enhancements affecting both the quantity and the quality of the signal (see Methods). **(E)** Same as (C) but using parameters that represent future enhancements.

In order to investigate how well can phylogenetic analysis of somatic mutations be used for answering such questions we simulated lineage trees consisting of two subclones, which have a common ancestor of a specific distance (Fig 3A). We then estimated the distance within and between the two cell groups and performed a statistical test to check whether the two cell groups are separated (see Methods). Fig 3B shows a similar heatmap to Fig 2B but presents the probability of identifying that the two cell groups are independent, using X = 2 and Y = 20 (see Fig 2A). Fig 3C presents the cost-optimized combinations for various values of X and Y. Fig 3D and 3E show the performance using enhanced parameters that correspond to future enhancements. Results for the other STR mutation rates and SNV are presented in S2 File.

## Discussion

During normal mitotic cell division DNA is replicated with very high, but not absolute, precision, which leads to the incorporation of somatic mutations. These somatic mutations accumulated since the zygotic stage, endow each cell in our bodies with a genomic signature that is unique with a very high probability [17]. Sequencing cell bulks for somatic mutations may supply a coarse estimation of the cell population distribution but cannot specify the deterministic position in the lineage tree of each cell and uncover population heterogeneity. Advancements in single cell genomics offer a unique opportunity to detect somatic mutations private to each cell and use them to understand the underlying dynamics of cell lineages with high precision. Unfortunately, sequencing accurately the entire genome of each single cell is still prohibitively expensive and technically challenging. In recent years there have been several attempts to use single cells genomic data in order to uncover various lineage dynamics. These attempts included SC whole genome sequencing [26], exome sequencing [5], and genotyping of targeted loci [6], or combinations of thereof [2]. There is a tradeoff between genomic coverage and sample density and the question of finding their right quantity and balance depends on parameters such as cost, technical constraints and the specifics of the lineage question. In this paper we offer a framework for answering this question by modeling and simulating the entire process of lineage analysis taking into consideration the different aspects of SC genomics analysis, calibrated using real experiments, and possible lineage dynamics. The suggested framework can help researchers in planning and optimizing their lineage experiments and can also point out experimental aspects that should be improved in order to increase the chances for meaningful outcomes. We selected a basic triplet tree structure and two aspects of lineage questions that are widely tackled, namely identifying depth differences and identifying independent clusters, and mapped the feasibility of answering them using a wide variety of parameters, including different mutation types, different mutation rates and various combinations of distances between the cell groups. The results can serve as a guideline for planning a lineage experiment or as a reference point for tailoring a solution for a more specific setting. Future experiments can help in fine-tuning the different modeling aspects, such as ADO, noise and possible lineage scenarios. Furthermore, these aspects can also be updated as new and more advanced biochemical protocols, technological or computational tools are developed.

## Methods

### Mutational Models

STR mutations were modeled using the single-step model (SSM) [22]. For each STR loci of length *x*, its length is updated during each cell division using the following function:

$$f_{STR}(x)=\left\{ \begin{array}{l} \begin{array}{l} x+1\;with\;probability\frac{p_{STR}}{2}\\ x-1\;with\;probability\frac{p_{STR}}{2} \end{array}\\ x\;otherwise \end{array}\right.$$

where *p*_*STR*_ is the mutation probability. In this paper we used three mutation scales, namely 10^−3^, 10^−4^, 10^−5^, corresponding to possible STR mutations rates. We note that some STRs can display more complex mutational patterns; however, the SSM is the most common model used and constitutes a good approximation.

SNV mutations were modeled by randomly changing each base with probability *p*_*SNV*_ during each cell division, where we used three mutations scales, 10^−7^, 10^−8^, 10^−9^.

We note that most chromosomes, except for the X and Y chromosomes in males, have two copies. This may introduce additional complexity to the analysis of SC genomic loci since a mutation can occur in one copy or the other. However, for MS loci this can be overcome by analyzing only sex chromosomes of males [6, 9, 10] or by analyzing loci with heterogeneous alleles that contain MS with different repeat number [27]. As for SNV analysis, the probability of a double mutation is low enough in order to allow a unique identification of random somatic mutations in each locus. Since the *ex-vivo* experimental data that we used in order to model the ADO and the noise of the SC genomic signal included mostly data from the X chromosome, we opted to analyze the simplified single allele scenario in this work. However, we are currently working on computational methods for analyzing biallelic signal, which will allow analyzing signal from autosomes and will also enable to extend the results presented here for more complex scenarios.

### ADO Modeling

Since a human cell contains only one copy of a diploid genome there is a big chance that some parts of the DNA will be damaged or lost during the different amplification stages. Because of the stochastic nature of the amplification, one could also expect a relatively large variability in the amplification quality of different samples. In addition, there could be amplification biases where some parts of the genome are better amplified than others, resulting in some loci having a better chance to be detected. In order to simulate the dropout patterning of the experimental data we sought to find a modeling approach that will mimic the real behavior as much as possible. The experimental data evidently show that the allelic dropout is not random but is dependent on both the sample quality and the genomic location. In order to capture the variability of the signal quality in both the individual samples and the different loci we modeled the allelic dropout of single cell DNA samples by assigning distinct dropout probabilities for each sample and for each locus. Given *M* individual samples and *N* loci we define the *mutation table T* = {*t*_*ij*_: *i* = 1.*M*, *j* = 1.*N*} such that *t*_*ij*_ equals the mutation call of sample *i* at locus *j*. In the case of allelic dropout we set *t*_*ij*_ = ∅. We define the *mutation signal table* as *X* = {*x*_*ij*_: *i* = 1.*M*, *j* = 1.*N*}, where

$$x_{ij}=\left\{ \begin{array}{l} 0\;if\;t_{ij}=\emptyset \\ 1\;otherwise \end{array}\right.$$

We define *P* = (*p*_*i*_: *i* = 1.*M*) as the probability of obtaining a signal in each sample and *Q* = (*q*_*j*_: *j* = 1.*N*) as the probability of obtaining a signal in each locus. The probability of obtaining a signal in sample *i* and locus *j* thus equals *p*_*i*_*q*_*j*_.

In order to estimate these probabilities using the real *ex-vivo* data, we used a Maximum Likelihood (ML) approach. Given the mutation signal table data *X* = {*x*_*ij*_}, the log likelihood is:

$$logL(P,Q;X)\;\propto \;logP(X|P,Q)=\sum_{i=1}^{M}\sum_{j=1}^{N}{log(x}_{ij}p_{i}q_{j}+(1-x_{ij})(1-p_{i}q_{j}))$$

The ML estimator of *P* and *Q* is thus:

$$\underset{P,Q}{\mathrm{argmax}}\left(log(L(P,Q;X))\right)$$

We approximated the solution using simulated annealing and validated the results by repeating the procedure with various starting points. For the data *X* we used an *ex-vivo* experiment in which 167 single cells were amplified and analyzed for their genomic signal. For prediction of future enhancement we used the calculated probabilities *p*,*q* and increased their relative value by 25%.

### Noise Modeling

Noise modeling differs between STR and SNV because STRs are much more prone to errors introduced during the amplification stages. For STR mutations we defined noise as the probability for each locus to randomly shift by one repeat unit compared to its true value. In order to estimate this probability we used the analysis results of duplicate cells from an *ex-vivo* experiment and measured the rate of inconsistency between supposedly identical genomes.

For SNV mutations we set the probability for noise to be 10^−4^ as measured using SC calling results of next-generation sequencing data [28].

For prediction of future enhancement we used the noise probability value divided by 2.

### Cost of Analysis

We have divided the analysis cost into two parts, namely, the overhead of analyzing a single cell and the analysis cost per single locus. A detailed cost analysis is not presented in this manuscript, however, an approximation for a complete analysis of a single cell is 30$, from which 10$ are considered to be fixed overhead and 20$ are used for analyzing either 2000 STR loci or 20,000 single bases. We thus approximated the analysis cost of a single STR locus to be 20/2000 = 0.01$ and the analysis cost of a single base (SNV) to be 20/20,000 = 0.0001$.

In order to calculate the cost as presented in Figs 2 and 3 we used the following function:

$$f_{Cost}=CostLoc\;*\;x+CostSamp\;*\;y$$

where *CostLoc* = 0.01 for STR and 0.0001 for SNV, *CostSamp* = 10, *x* = # *of loci*, *y* = # *of samples* and *x*,*y* are constrained to the white line in Figs 2 and 3 (corresponding to success probability of 95%). Minimal cost is obtained by finding the minimum of *f*_*Cost*_.

### Tree Reconstruction Algorithm

For the triplet trees reconstruction we used the Neighbor-Joining (NJ) algorithm [29] with the absolute distance function:

Given a mutation table $T=\{T_{i}^{l};i=1..M,l=1..N\}$, with *M* samples and *N* loci, where $T_{i}^{l}$ is the genotyping of locus *l* in sample *i*, the distance between each two samples is:

$$D(i,j)=\frac{1}{N}\sum_{l=1}^{N}\left|T_{i}^{l}-T_{j}^{l}\right|$$

where only loci with signal in both samples are counted. For the three example samples with the following 5 loci genotype:

$$T_{1}=(10,\emptyset ,\emptyset ,8,12)$$

$$T_{2}=(12,\emptyset ,7,8,\emptyset )$$

$$T_{3}=(10,\emptyset ,7,8,11)$$

where ∅ means that there is no signal in that locus, the distances are:

$$D(1,2)=\frac{1}{2}(\left|10-11|+|8-8\right|)=\frac{1}{2}(1+0)=\frac{1}{2}$$

$$D(1,3)=\frac{1}{2}(\left|10-12|+|8-8\right|)=\frac{1}{2}(2+0)=1$$

$$D(2,3)=\frac{1}{3}(\left|12-10|+|7-7|+|8-8\right|)=\frac{1}{3}(2+0+0)=\frac{2}{3}$$

The result of the NJ tree reconstruction algorithm on these samples is depicted in S1 Fig.

We note that alternatives to distance-based methods for phylogeny estimation exist, which might yield better results or improve the cost efficiency; however, analyzing or developing such methods is not in the scope of this paper and is a subject of an ongoing research in our lab.

### Measuring Significant Depth Differences

Given two groups of cells *A* = {*a*_*i*_} and *B* = {*b*_*j*_} we define a binary classifier *f* that decides whether there is a depth difference between them or not. We define *D*(*x*) as the distance between the cell *x* and the root of the tree where *D* is calculated using the absolute distance function as defined above. We define the set *D*(*X*) = {*D*(*x*)}_*x*∈*X*_ where *X* is a group of cells. We define *ttest*(*D*(*A*),*D*(*B*)) as the p-value obtained from a t-test between the set of distances *D*(*A*) and *D*(*B*). The classifier *f* is defined as follows:

$$f(A,B)=\left\{ \begin{array}{l} 1\;if\;ttest(D(A),D(B))\leq 0.05\\ 0\;otherwise \end{array}\right.$$

where *f*(*A*,*B*) = 1 means that there is a significant distance between the cell groups *A* and *B*.

In the words of hypothesis testing, if we define the null hypothesis to be that there is no depth difference between *A* and *B* then from the definition of *f* if the depth of the two populations is equally distributed the probability of incorrectly rejecting the null hypothesis, i.e., the type I error, is 5% and the statistical power is depicted in Fig 2B.

### Measuring Significant Independent Clustering

Similarly to the case of depth differences, we define *D*(*x*,*y*) as the distance between cell *x* and cell *y*, and *D*(*X*,*Y*) = {*D*(*x*,*y*)}_*x*∈*X*,*y*∈*Y*_. We define the clustering classifier *f* to be:

$$f(A,B)=\left\{ \begin{array}{l} 1\;if\;ttest(D(A,A),D(A,B))\leq 0.05\\ 0\;otherwise \end{array}\right.$$

i.e., we measure the difference in the average distance of cells within the group *A* and the distance of cells between group *A* and group *B*. Similarly to the case of the depth differences, the type I error is 5% and the statistical power is depicted in Fig 3B.

We note that the measures presented here for identifying significant depth differences and clustering are used for proof of concept and there may be better ones. However, finding better measures is not in the scope of this paper and is a subject of future research.

## Supporting Information

S1 File eSTG programs of the simulated lineage trees. (ZIP)

S2 File Results of cell lineage analysis. Results include figures similar to Figs 1B, 2B and 3B but using STR mutations with mutation rates 10^−3^ and 10^−5^ and SNV mutations with mutation rates 10^−7^, 10^−8^, 10^−9^ using current and improved values for ADO and noise corresponding to future quality enhancements of single cell genomics.
(ZIP)

S1 Fig Example of NJ tree reconstruction of a triplet. (TIF)
