# Supplementary figures and images for: Accuracy of Answers to Cell Lineage Questions Depends on Single-Cell Genomics Data Quality and Quantity (part 1 of 3)
Source: PLoS Comput Biol. 2016 Jun 13;12(6):e1004983. doi: 10.1371/journal.pcbi.1004983 (PMC4905655; doi:10.1371/journal.pcbi.1004983)

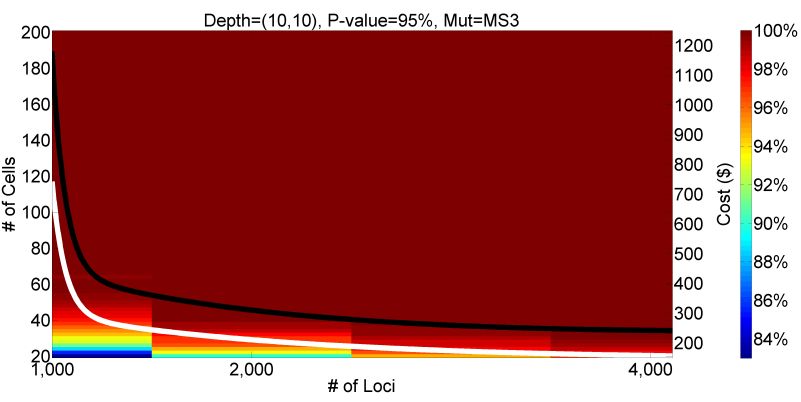

Supplement: S2 File — Results include figures similar to Figs 1B, 2B and 3B but using STR mutations with mutation rates 10−3 and 10−5 and SNV mutations with mutation rates 10−7, 10−8, 10−9 using current and improved values for ADO and noise corresponding to future quality enhancements of single cell genomics. (ZIP) [file pcbi.1004983.s002.zip › Clusters/Clusters_Param_10_10_pval_95_MS3_Res.jpg]

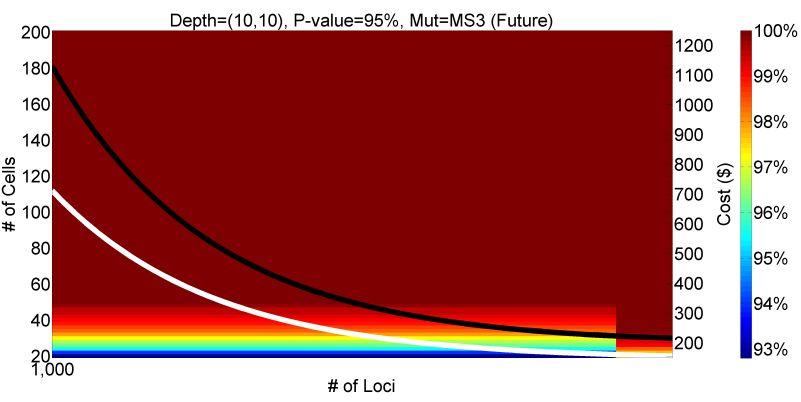

Supplement: S2 File — Results include figures similar to Figs 1B, 2B and 3B but using STR mutations with mutation rates 10−3 and 10−5 and SNV mutations with mutation rates 10−7, 10−8, 10−9 using current and improved values for ADO and noise corresponding to future quality enhancements of single cell genomics. (ZIP) [file pcbi.1004983.s002.zip › Clusters/Clusters_Param_10_10_pval_95_MS3_Res_Future.jpg]

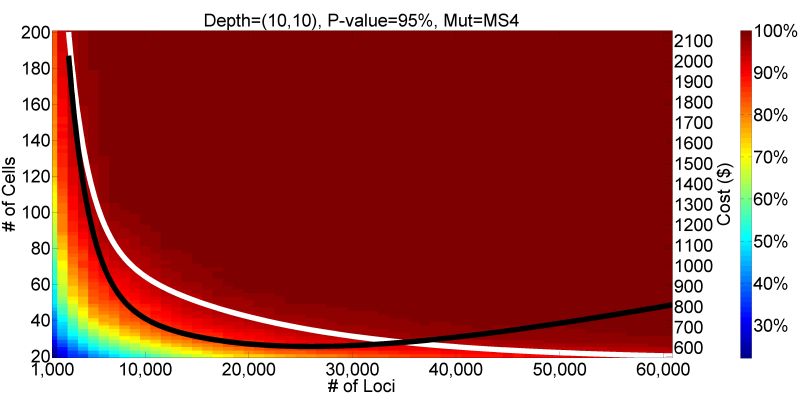

Supplement: S2 File — Results include figures similar to Figs 1B, 2B and 3B but using STR mutations with mutation rates 10−3 and 10−5 and SNV mutations with mutation rates 10−7, 10−8, 10−9 using current and improved values for ADO and noise corresponding to future quality enhancements of single cell genomics. (ZIP) [file pcbi.1004983.s002.zip › Clusters/Clusters_Param_10_10_pval_95_MS4_Res.jpg]

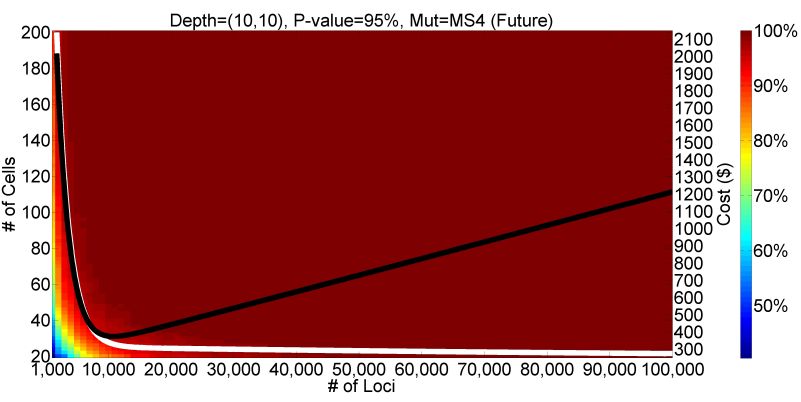

Supplement: S2 File — Results include figures similar to Figs 1B, 2B and 3B but using STR mutations with mutation rates 10−3 and 10−5 and SNV mutations with mutation rates 10−7, 10−8, 10−9 using current and improved values for ADO and noise corresponding to future quality enhancements of single cell genomics. (ZIP) [file pcbi.1004983.s002.zip › Clusters/Clusters_Param_10_10_pval_95_MS4_Res_Future.jpg]

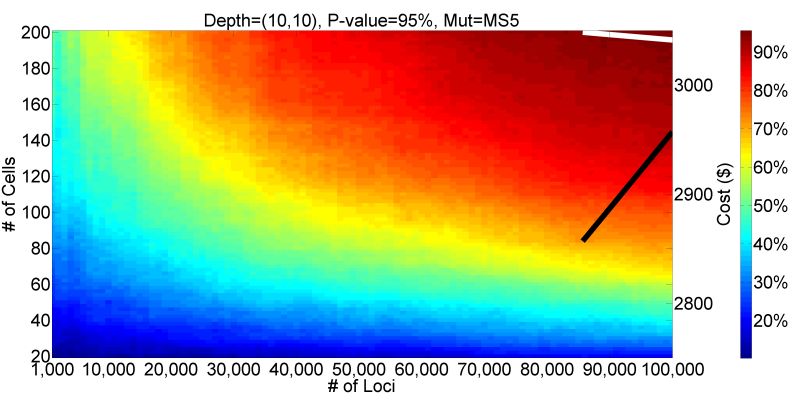

Supplement: S2 File — Results include figures similar to Figs 1B, 2B and 3B but using STR mutations with mutation rates 10−3 and 10−5 and SNV mutations with mutation rates 10−7, 10−8, 10−9 using current and improved values for ADO and noise corresponding to future quality enhancements of single cell genomics. (ZIP) [file pcbi.1004983.s002.zip › Clusters/Clusters_Param_10_10_pval_95_MS5_Res.jpg]

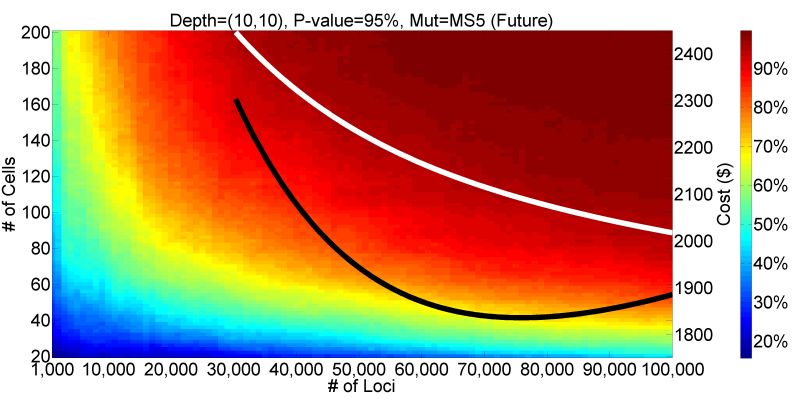

Supplement: S2 File — Results include figures similar to Figs 1B, 2B and 3B but using STR mutations with mutation rates 10−3 and 10−5 and SNV mutations with mutation rates 10−7, 10−8, 10−9 using current and improved values for ADO and noise corresponding to future quality enhancements of single cell genomics. (ZIP) [file pcbi.1004983.s002.zip › Clusters/Clusters_Param_10_10_pval_95_MS5_Res_Future.jpg]

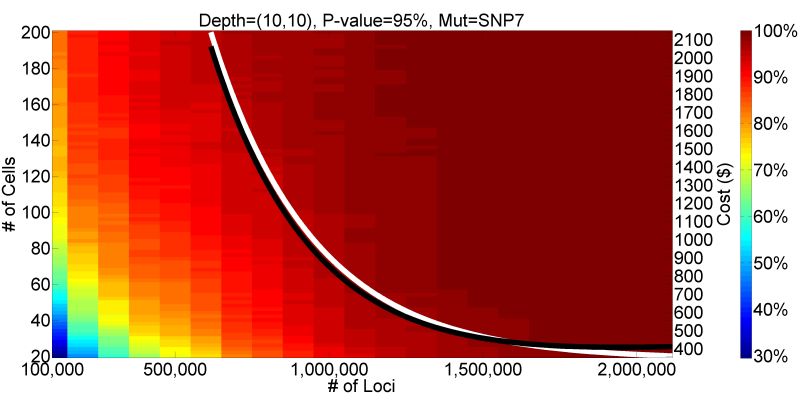

Supplement: S2 File — Results include figures similar to Figs 1B, 2B and 3B but using STR mutations with mutation rates 10−3 and 10−5 and SNV mutations with mutation rates 10−7, 10−8, 10−9 using current and improved values for ADO and noise corresponding to future quality enhancements of single cell genomics. (ZIP) [file pcbi.1004983.s002.zip › Clusters/Clusters_Param_10_10_pval_95_SNP7_Res.jpg]

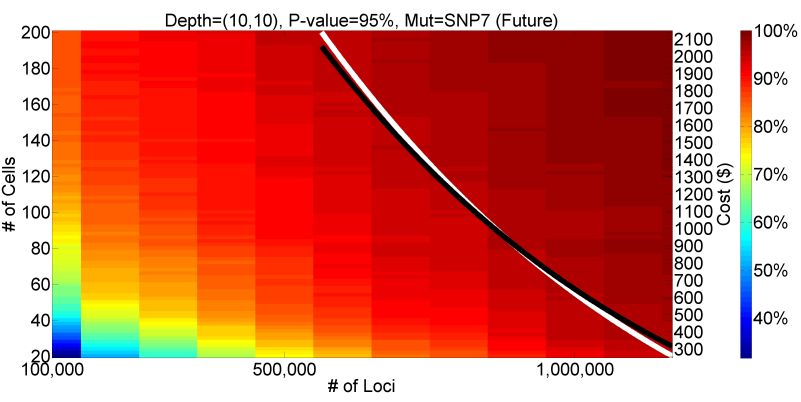

Supplement: S2 File — Results include figures similar to Figs 1B, 2B and 3B but using STR mutations with mutation rates 10−3 and 10−5 and SNV mutations with mutation rates 10−7, 10−8, 10−9 using current and improved values for ADO and noise corresponding to future quality enhancements of single cell genomics. (ZIP) [file pcbi.1004983.s002.zip › Clusters/Clusters_Param_10_10_pval_95_SNP7_Res_Future.jpg]

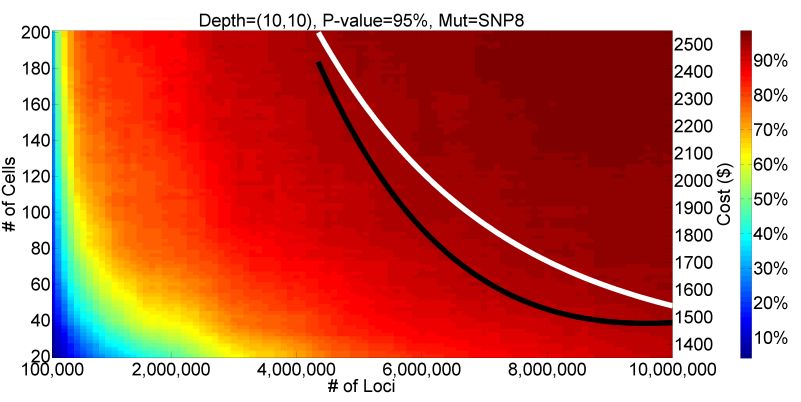

Supplement: S2 File — Results include figures similar to Figs 1B, 2B and 3B but using STR mutations with mutation rates 10−3 and 10−5 and SNV mutations with mutation rates 10−7, 10−8, 10−9 using current and improved values for ADO and noise corresponding to future quality enhancements of single cell genomics. (ZIP) [file pcbi.1004983.s002.zip › Clusters/Clusters_Param_10_10_pval_95_SNP8_Res.jpg]

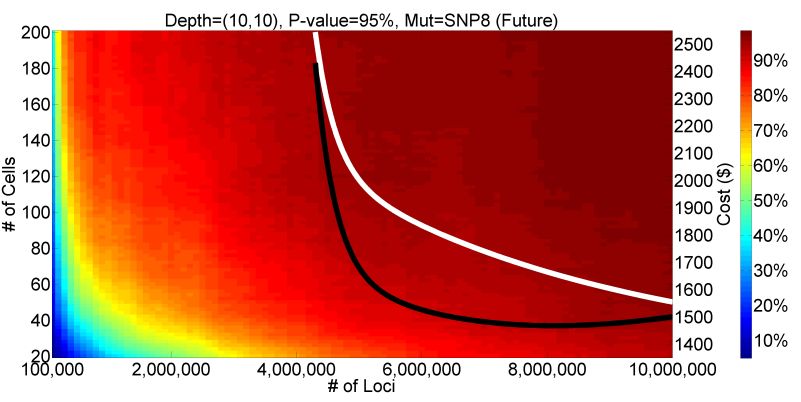

Supplement: S2 File — Results include figures similar to Figs 1B, 2B and 3B but using STR mutations with mutation rates 10−3 and 10−5 and SNV mutations with mutation rates 10−7, 10−8, 10−9 using current and improved values for ADO and noise corresponding to future quality enhancements of single cell genomics. (ZIP) [file pcbi.1004983.s002.zip › Clusters/Clusters_Param_10_10_pval_95_SNP8_Res_Future.jpg]

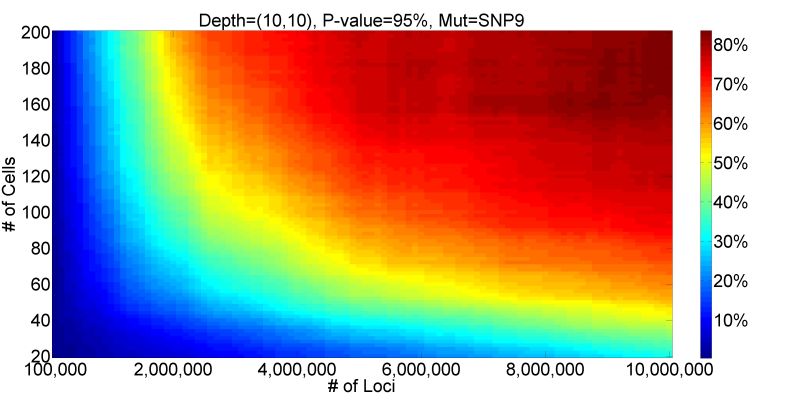

Supplement: S2 File — Results include figures similar to Figs 1B, 2B and 3B but using STR mutations with mutation rates 10−3 and 10−5 and SNV mutations with mutation rates 10−7, 10−8, 10−9 using current and improved values for ADO and noise corresponding to future quality enhancements of single cell genomics. (ZIP) [file pcbi.1004983.s002.zip › Clusters/Clusters_Param_10_10_pval_95_SNP9_Res.jpg]

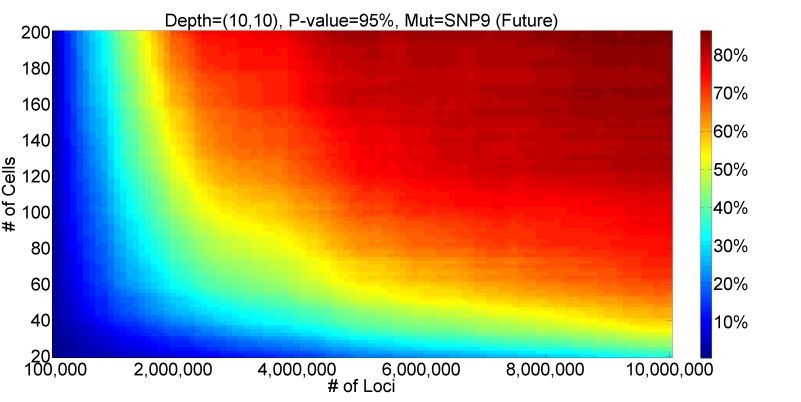

Supplement: S2 File — Results include figures similar to Figs 1B, 2B and 3B but using STR mutations with mutation rates 10−3 and 10−5 and SNV mutations with mutation rates 10−7, 10−8, 10−9 using current and improved values for ADO and noise corresponding to future quality enhancements of single cell genomics. (ZIP) [file pcbi.1004983.s002.zip › Clusters/Clusters_Param_10_10_pval_95_SNP9_Res_Future.jpg]

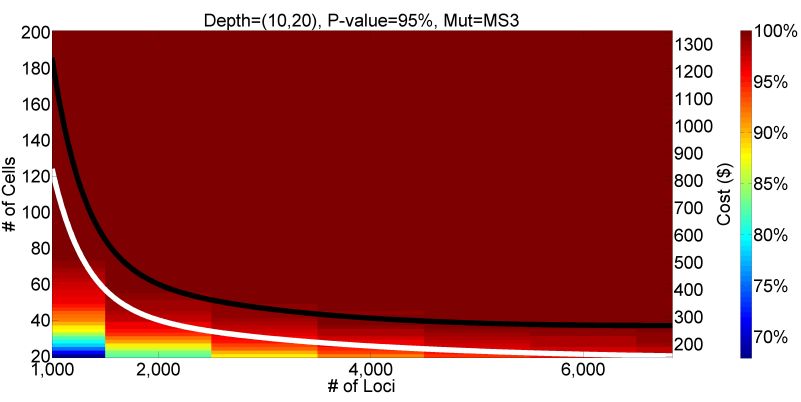

Supplement: S2 File — Results include figures similar to Figs 1B, 2B and 3B but using STR mutations with mutation rates 10−3 and 10−5 and SNV mutations with mutation rates 10−7, 10−8, 10−9 using current and improved values for ADO and noise corresponding to future quality enhancements of single cell genomics. (ZIP) [file pcbi.1004983.s002.zip › Clusters/Clusters_Param_10_20_pval_95_MS3_Res.jpg]

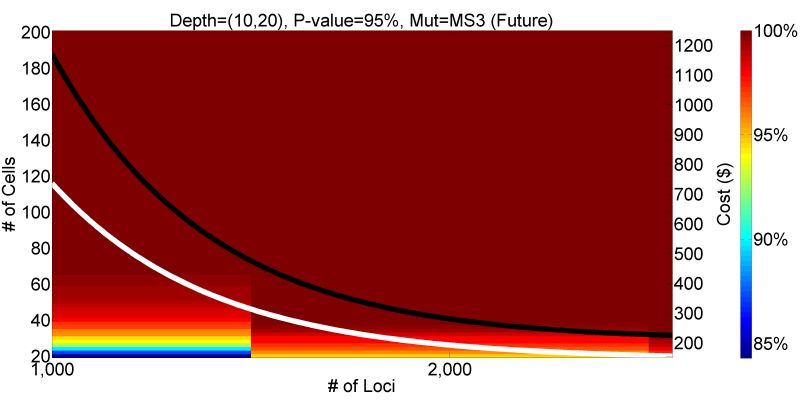

Supplement: S2 File — Results include figures similar to Figs 1B, 2B and 3B but using STR mutations with mutation rates 10−3 and 10−5 and SNV mutations with mutation rates 10−7, 10−8, 10−9 using current and improved values for ADO and noise corresponding to future quality enhancements of single cell genomics. (ZIP) [file pcbi.1004983.s002.zip › Clusters/Clusters_Param_10_20_pval_95_MS3_Res_Future.jpg]

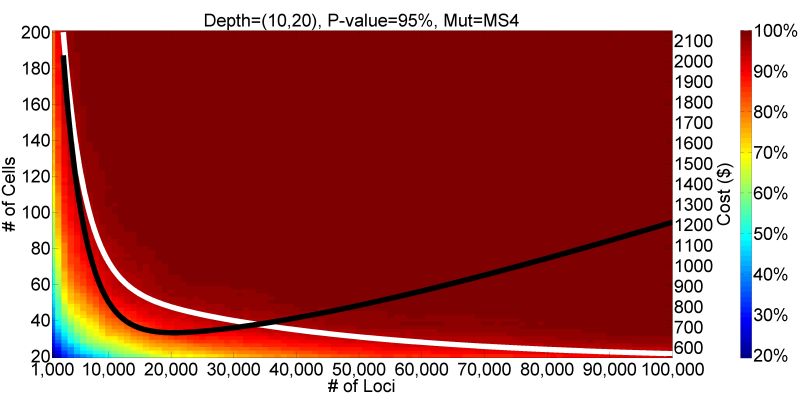

Supplement: S2 File — Results include figures similar to Figs 1B, 2B and 3B but using STR mutations with mutation rates 10−3 and 10−5 and SNV mutations with mutation rates 10−7, 10−8, 10−9 using current and improved values for ADO and noise corresponding to future quality enhancements of single cell genomics. (ZIP) [file pcbi.1004983.s002.zip › Clusters/Clusters_Param_10_20_pval_95_MS4_Res.jpg]

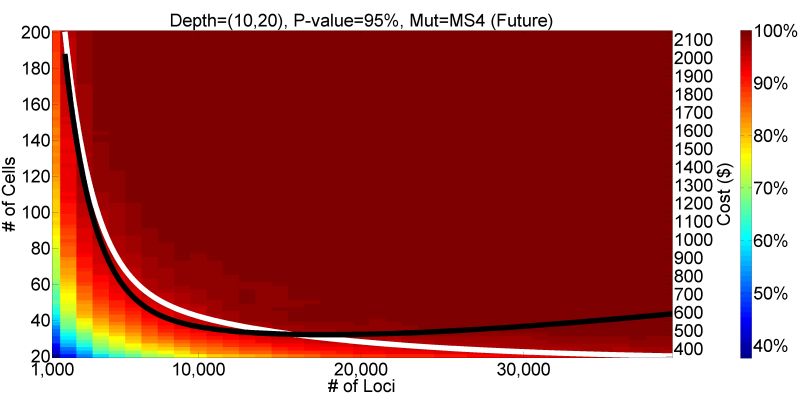

Supplement: S2 File — Results include figures similar to Figs 1B, 2B and 3B but using STR mutations with mutation rates 10−3 and 10−5 and SNV mutations with mutation rates 10−7, 10−8, 10−9 using current and improved values for ADO and noise corresponding to future quality enhancements of single cell genomics. (ZIP) [file pcbi.1004983.s002.zip › Clusters/Clusters_Param_10_20_pval_95_MS4_Res_Future.jpg]

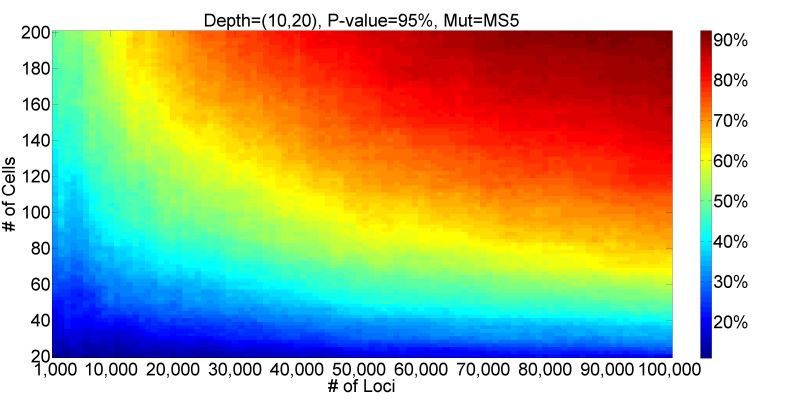

Supplement: S2 File — Results include figures similar to Figs 1B, 2B and 3B but using STR mutations with mutation rates 10−3 and 10−5 and SNV mutations with mutation rates 10−7, 10−8, 10−9 using current and improved values for ADO and noise corresponding to future quality enhancements of single cell genomics. (ZIP) [file pcbi.1004983.s002.zip › Clusters/Clusters_Param_10_20_pval_95_MS5_Res.jpg]

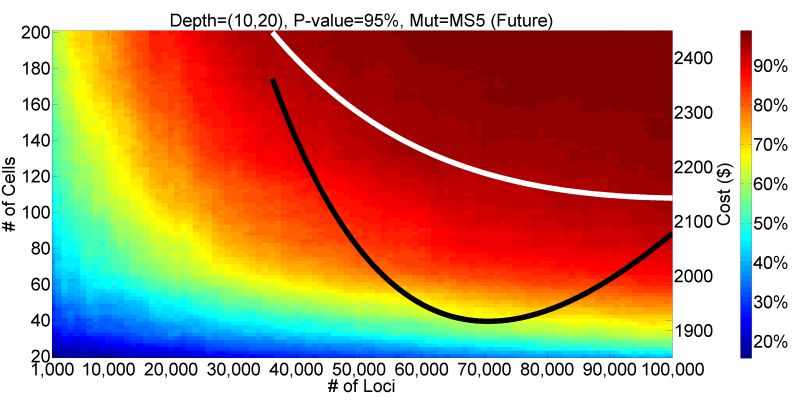

Supplement: S2 File — Results include figures similar to Figs 1B, 2B and 3B but using STR mutations with mutation rates 10−3 and 10−5 and SNV mutations with mutation rates 10−7, 10−8, 10−9 using current and improved values for ADO and noise corresponding to future quality enhancements of single cell genomics. (ZIP) [file pcbi.1004983.s002.zip › Clusters/Clusters_Param_10_20_pval_95_MS5_Res_Future.jpg]

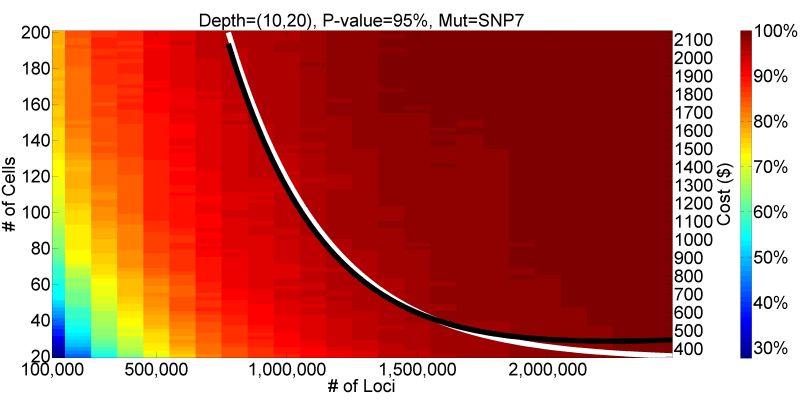

Supplement: S2 File — Results include figures similar to Figs 1B, 2B and 3B but using STR mutations with mutation rates 10−3 and 10−5 and SNV mutations with mutation rates 10−7, 10−8, 10−9 using current and improved values for ADO and noise corresponding to future quality enhancements of single cell genomics. (ZIP) [file pcbi.1004983.s002.zip › Clusters/Clusters_Param_10_20_pval_95_SNP7_Res.jpg]

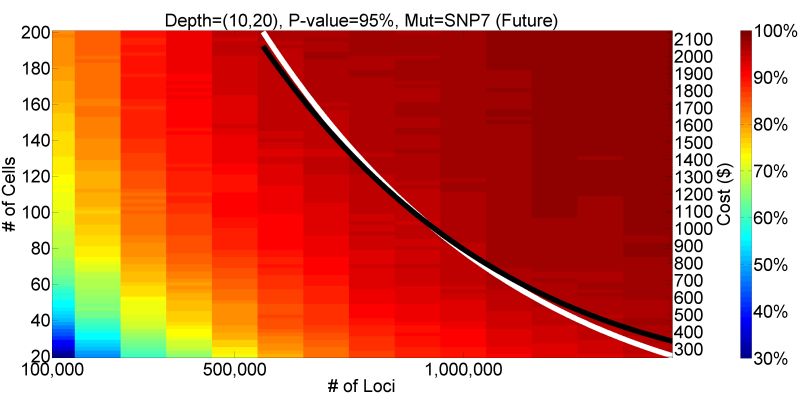

Supplement: S2 File — Results include figures similar to Figs 1B, 2B and 3B but using STR mutations with mutation rates 10−3 and 10−5 and SNV mutations with mutation rates 10−7, 10−8, 10−9 using current and improved values for ADO and noise corresponding to future quality enhancements of single cell genomics. (ZIP) [file pcbi.1004983.s002.zip › Clusters/Clusters_Param_10_20_pval_95_SNP7_Res_Future.jpg]

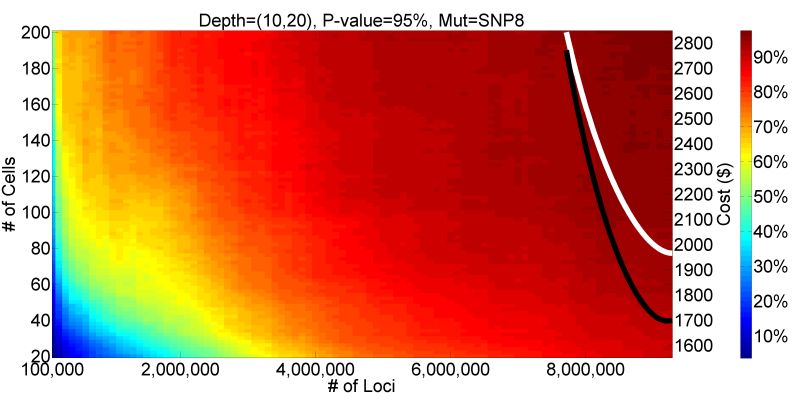

Supplement: S2 File — Results include figures similar to Figs 1B, 2B and 3B but using STR mutations with mutation rates 10−3 and 10−5 and SNV mutations with mutation rates 10−7, 10−8, 10−9 using current and improved values for ADO and noise corresponding to future quality enhancements of single cell genomics. (ZIP) [file pcbi.1004983.s002.zip › Clusters/Clusters_Param_10_20_pval_95_SNP8_Res.jpg]

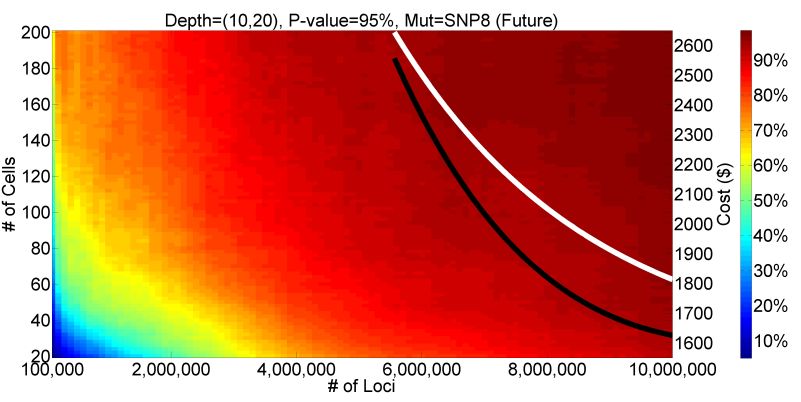

Supplement: S2 File — Results include figures similar to Figs 1B, 2B and 3B but using STR mutations with mutation rates 10−3 and 10−5 and SNV mutations with mutation rates 10−7, 10−8, 10−9 using current and improved values for ADO and noise corresponding to future quality enhancements of single cell genomics. (ZIP) [file pcbi.1004983.s002.zip › Clusters/Clusters_Param_10_20_pval_95_SNP8_Res_Future.jpg]

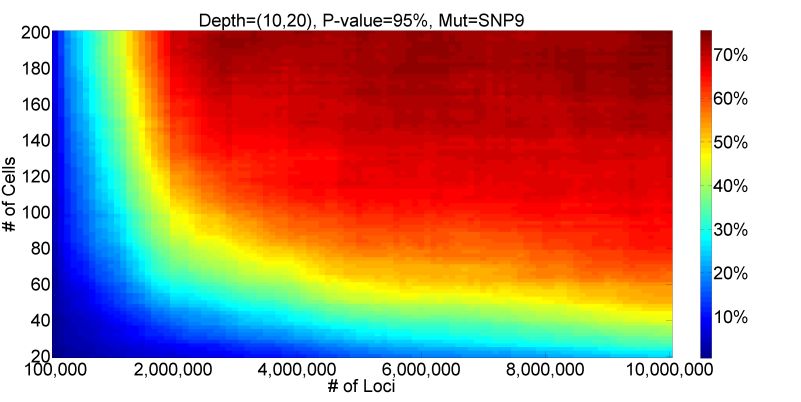

Supplement: S2 File — Results include figures similar to Figs 1B, 2B and 3B but using STR mutations with mutation rates 10−3 and 10−5 and SNV mutations with mutation rates 10−7, 10−8, 10−9 using current and improved values for ADO and noise corresponding to future quality enhancements of single cell genomics. (ZIP) [file pcbi.1004983.s002.zip › Clusters/Clusters_Param_10_20_pval_95_SNP9_Res.jpg]

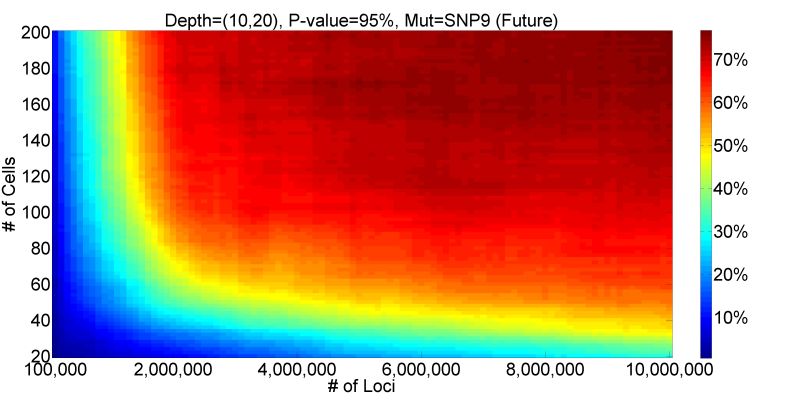

Supplement: S2 File — Results include figures similar to Figs 1B, 2B and 3B but using STR mutations with mutation rates 10−3 and 10−5 and SNV mutations with mutation rates 10−7, 10−8, 10−9 using current and improved values for ADO and noise corresponding to future quality enhancements of single cell genomics. (ZIP) [file pcbi.1004983.s002.zip › Clusters/Clusters_Param_10_20_pval_95_SNP9_Res_Future.jpg]

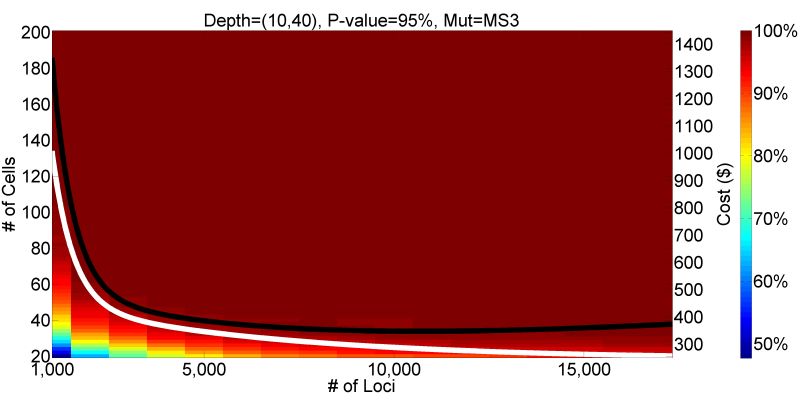

Supplement: S2 File — Results include figures similar to Figs 1B, 2B and 3B but using STR mutations with mutation rates 10−3 and 10−5 and SNV mutations with mutation rates 10−7, 10−8, 10−9 using current and improved values for ADO and noise corresponding to future quality enhancements of single cell genomics. (ZIP) [file pcbi.1004983.s002.zip › Clusters/Clusters_Param_10_40_pval_95_MS3_Res.jpg]

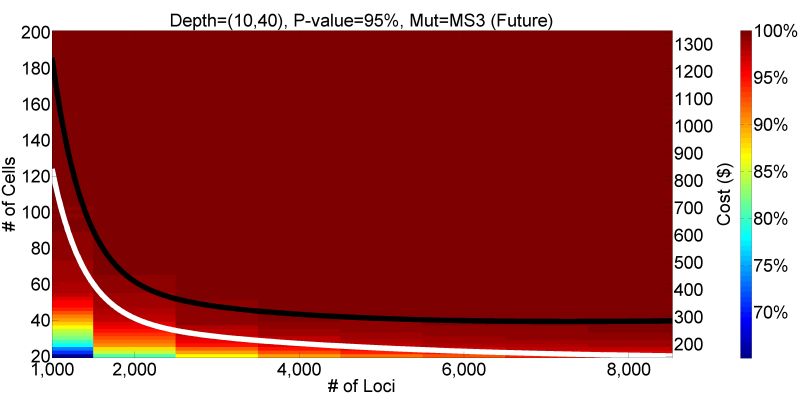

Supplement: S2 File — Results include figures similar to Figs 1B, 2B and 3B but using STR mutations with mutation rates 10−3 and 10−5 and SNV mutations with mutation rates 10−7, 10−8, 10−9 using current and improved values for ADO and noise corresponding to future quality enhancements of single cell genomics. (ZIP) [file pcbi.1004983.s002.zip › Clusters/Clusters_Param_10_40_pval_95_MS3_Res_Future.jpg]

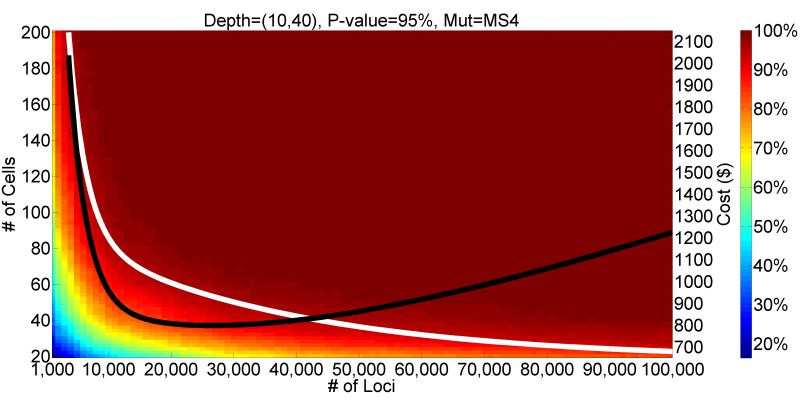

Supplement: S2 File — Results include figures similar to Figs 1B, 2B and 3B but using STR mutations with mutation rates 10−3 and 10−5 and SNV mutations with mutation rates 10−7, 10−8, 10−9 using current and improved values for ADO and noise corresponding to future quality enhancements of single cell genomics. (ZIP) [file pcbi.1004983.s002.zip › Clusters/Clusters_Param_10_40_pval_95_MS4_Res.jpg]

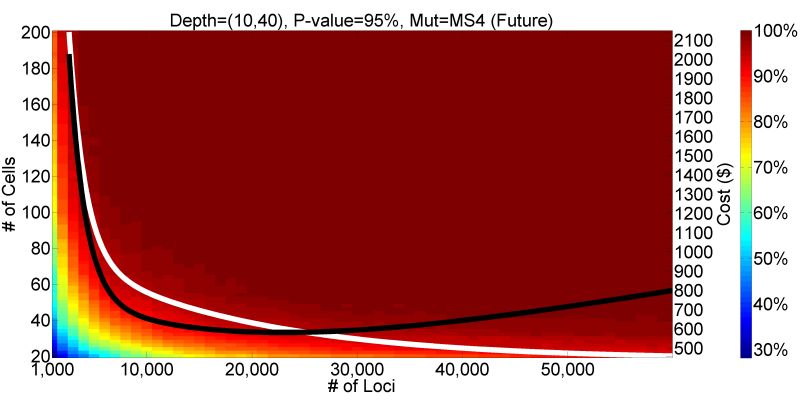

Supplement: S2 File — Results include figures similar to Figs 1B, 2B and 3B but using STR mutations with mutation rates 10−3 and 10−5 and SNV mutations with mutation rates 10−7, 10−8, 10−9 using current and improved values for ADO and noise corresponding to future quality enhancements of single cell genomics. (ZIP) [file pcbi.1004983.s002.zip › Clusters/Clusters_Param_10_40_pval_95_MS4_Res_Future.jpg]

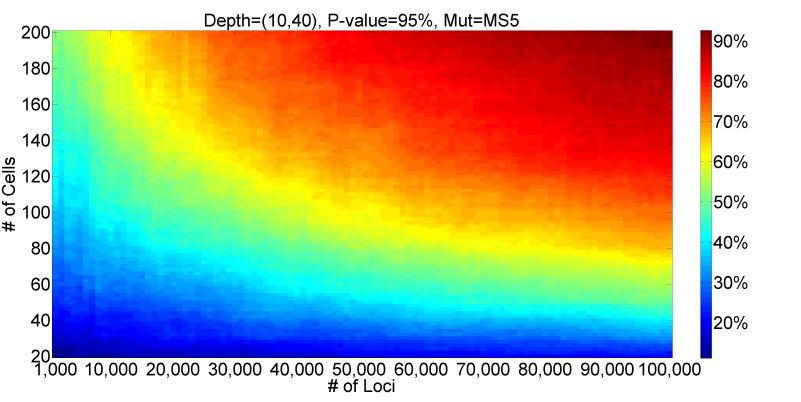

Supplement: S2 File — Results include figures similar to Figs 1B, 2B and 3B but using STR mutations with mutation rates 10−3 and 10−5 and SNV mutations with mutation rates 10−7, 10−8, 10−9 using current and improved values for ADO and noise corresponding to future quality enhancements of single cell genomics. (ZIP) [file pcbi.1004983.s002.zip › Clusters/Clusters_Param_10_40_pval_95_MS5_Res.jpg]

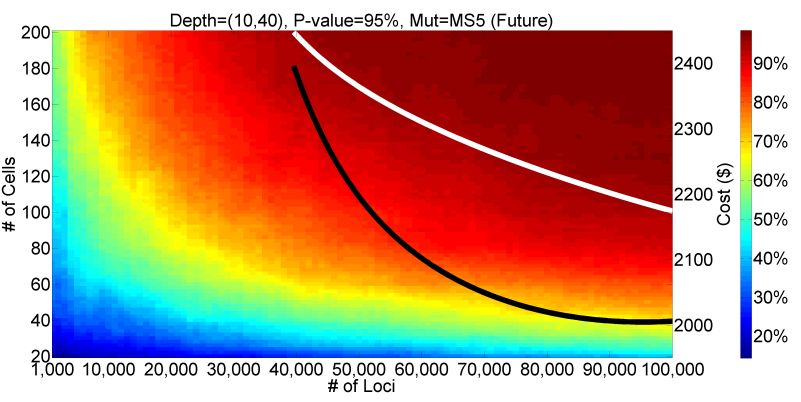

Supplement: S2 File — Results include figures similar to Figs 1B, 2B and 3B but using STR mutations with mutation rates 10−3 and 10−5 and SNV mutations with mutation rates 10−7, 10−8, 10−9 using current and improved values for ADO and noise corresponding to future quality enhancements of single cell genomics. (ZIP) [file pcbi.1004983.s002.zip › Clusters/Clusters_Param_10_40_pval_95_MS5_Res_Future.jpg]

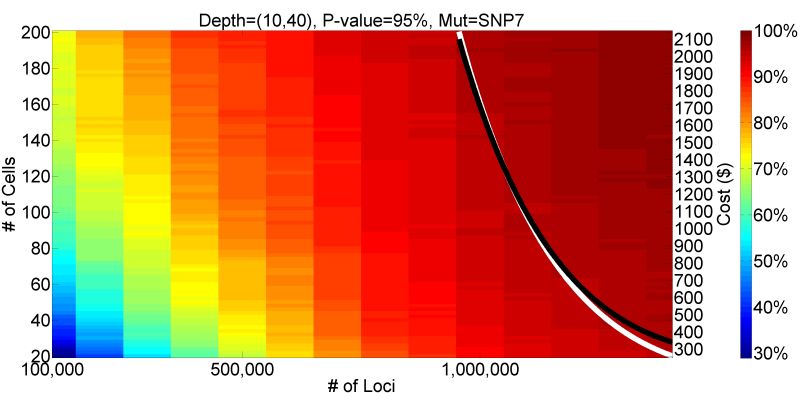

Supplement: S2 File — Results include figures similar to Figs 1B, 2B and 3B but using STR mutations with mutation rates 10−3 and 10−5 and SNV mutations with mutation rates 10−7, 10−8, 10−9 using current and improved values for ADO and noise corresponding to future quality enhancements of single cell genomics. (ZIP) [file pcbi.1004983.s002.zip › Clusters/Clusters_Param_10_40_pval_95_SNP7_Res.jpg]

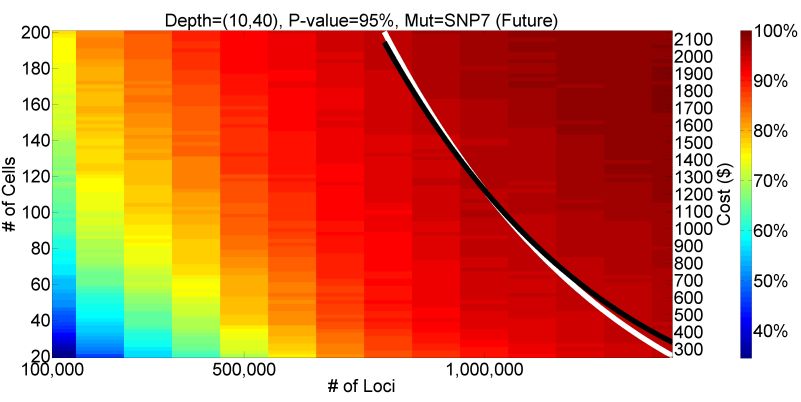

Supplement: S2 File — Results include figures similar to Figs 1B, 2B and 3B but using STR mutations with mutation rates 10−3 and 10−5 and SNV mutations with mutation rates 10−7, 10−8, 10−9 using current and improved values for ADO and noise corresponding to future quality enhancements of single cell genomics. (ZIP) [file pcbi.1004983.s002.zip › Clusters/Clusters_Param_10_40_pval_95_SNP7_Res_Future.jpg]

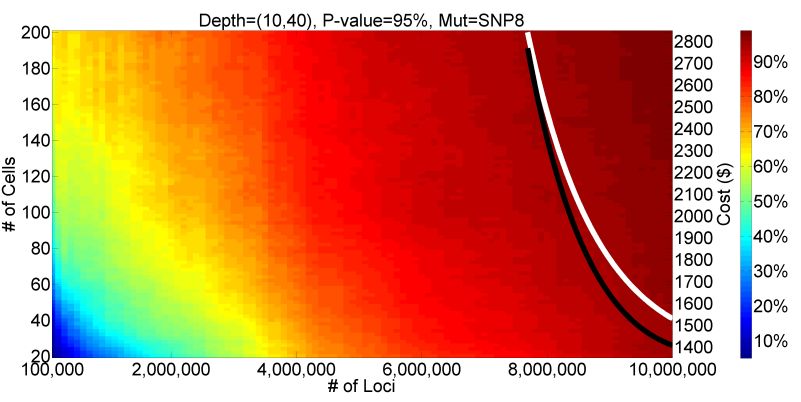

Supplement: S2 File — Results include figures similar to Figs 1B, 2B and 3B but using STR mutations with mutation rates 10−3 and 10−5 and SNV mutations with mutation rates 10−7, 10−8, 10−9 using current and improved values for ADO and noise corresponding to future quality enhancements of single cell genomics. (ZIP) [file pcbi.1004983.s002.zip › Clusters/Clusters_Param_10_40_pval_95_SNP8_Res.jpg]

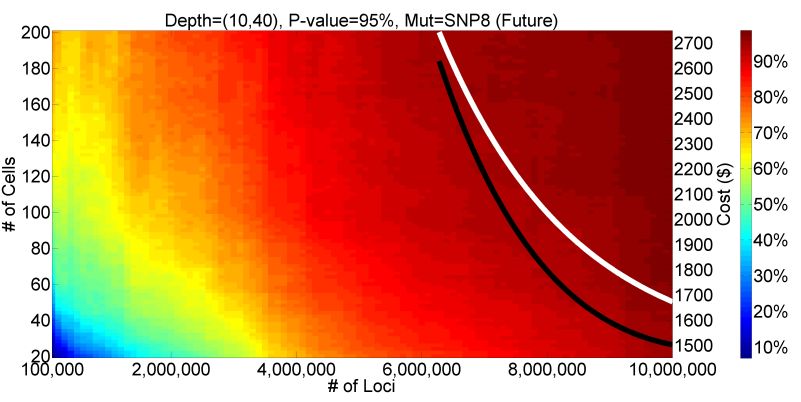

Supplement: S2 File — Results include figures similar to Figs 1B, 2B and 3B but using STR mutations with mutation rates 10−3 and 10−5 and SNV mutations with mutation rates 10−7, 10−8, 10−9 using current and improved values for ADO and noise corresponding to future quality enhancements of single cell genomics. (ZIP) [file pcbi.1004983.s002.zip › Clusters/Clusters_Param_10_40_pval_95_SNP8_Res_Future.jpg]

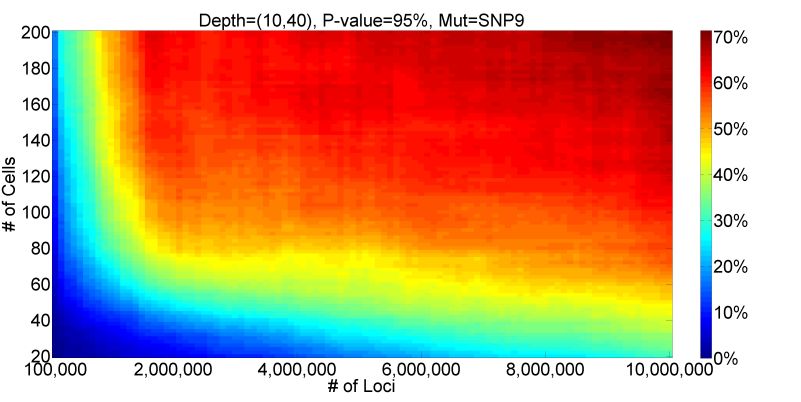

Supplement: S2 File — Results include figures similar to Figs 1B, 2B and 3B but using STR mutations with mutation rates 10−3 and 10−5 and SNV mutations with mutation rates 10−7, 10−8, 10−9 using current and improved values for ADO and noise corresponding to future quality enhancements of single cell genomics. (ZIP) [file pcbi.1004983.s002.zip › Clusters/Clusters_Param_10_40_pval_95_SNP9_Res.jpg]

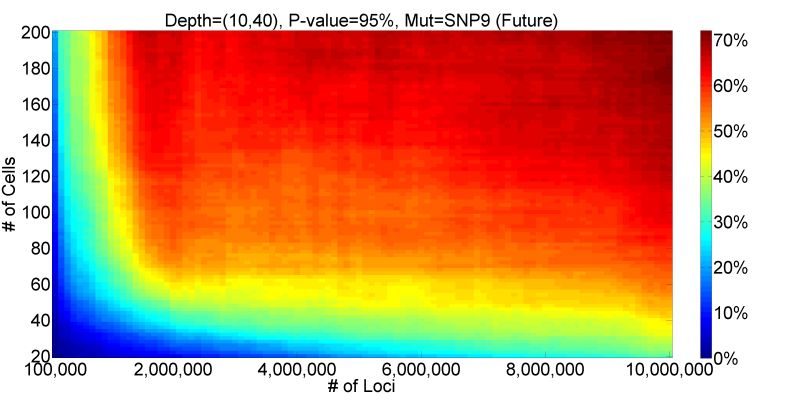

Supplement: S2 File — Results include figures similar to Figs 1B, 2B and 3B but using STR mutations with mutation rates 10−3 and 10−5 and SNV mutations with mutation rates 10−7, 10−8, 10−9 using current and improved values for ADO and noise corresponding to future quality enhancements of single cell genomics. (ZIP) [file pcbi.1004983.s002.zip › Clusters/Clusters_Param_10_40_pval_95_SNP9_Res_Future.jpg]

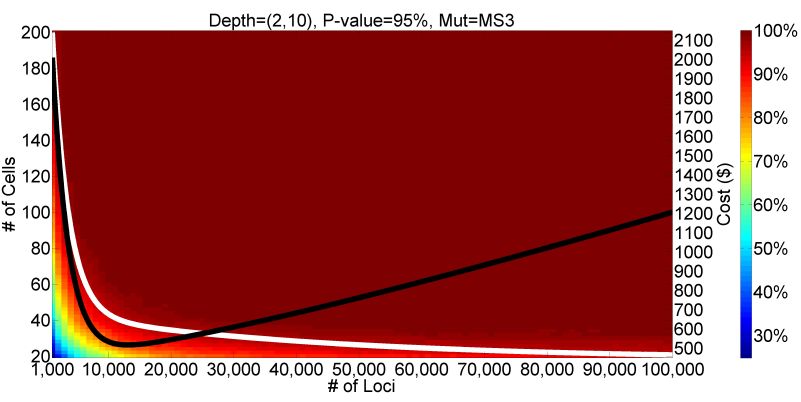

Supplement: S2 File — Results include figures similar to Figs 1B, 2B and 3B but using STR mutations with mutation rates 10−3 and 10−5 and SNV mutations with mutation rates 10−7, 10−8, 10−9 using current and improved values for ADO and noise corresponding to future quality enhancements of single cell genomics. (ZIP) [file pcbi.1004983.s002.zip › Clusters/Clusters_Param_2_10_pval_95_MS3_Res.jpg]

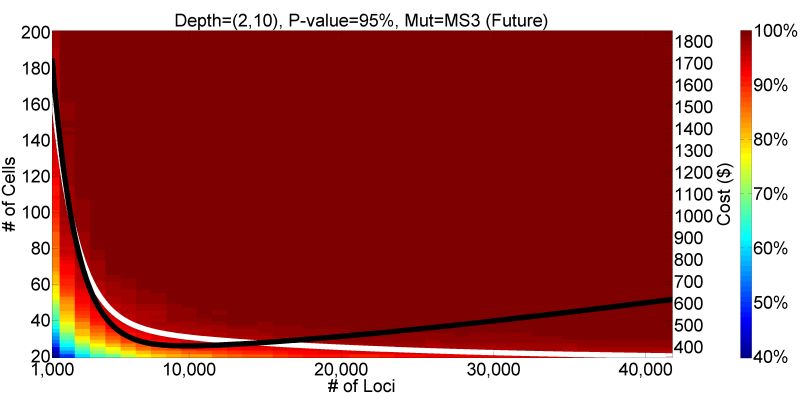

Supplement: S2 File — Results include figures similar to Figs 1B, 2B and 3B but using STR mutations with mutation rates 10−3 and 10−5 and SNV mutations with mutation rates 10−7, 10−8, 10−9 using current and improved values for ADO and noise corresponding to future quality enhancements of single cell genomics. (ZIP) [file pcbi.1004983.s002.zip › Clusters/Clusters_Param_2_10_pval_95_MS3_Res_Future.jpg]

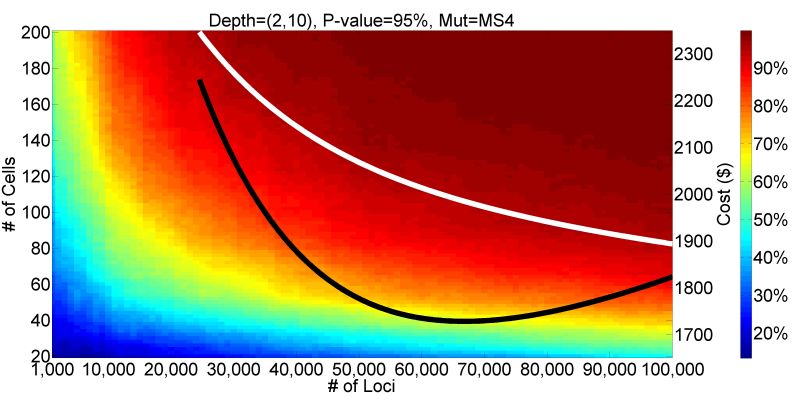

Supplement: S2 File — Results include figures similar to Figs 1B, 2B and 3B but using STR mutations with mutation rates 10−3 and 10−5 and SNV mutations with mutation rates 10−7, 10−8, 10−9 using current and improved values for ADO and noise corresponding to future quality enhancements of single cell genomics. (ZIP) [file pcbi.1004983.s002.zip › Clusters/Clusters_Param_2_10_pval_95_MS4_Res.jpg]

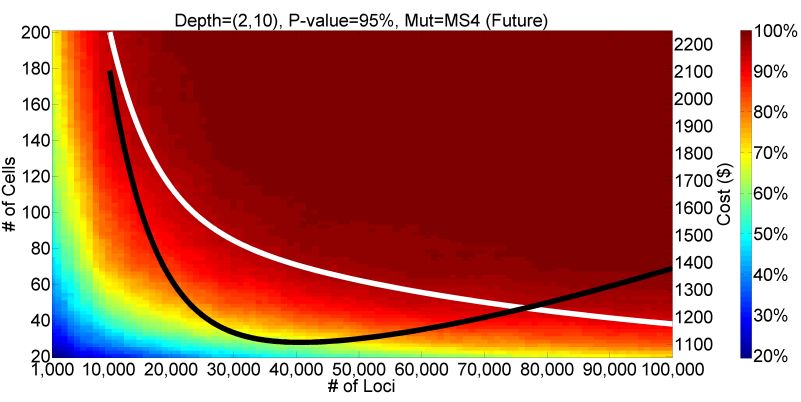

Supplement: S2 File — Results include figures similar to Figs 1B, 2B and 3B but using STR mutations with mutation rates 10−3 and 10−5 and SNV mutations with mutation rates 10−7, 10−8, 10−9 using current and improved values for ADO and noise corresponding to future quality enhancements of single cell genomics. (ZIP) [file pcbi.1004983.s002.zip › Clusters/Clusters_Param_2_10_pval_95_MS4_Res_Future.jpg]

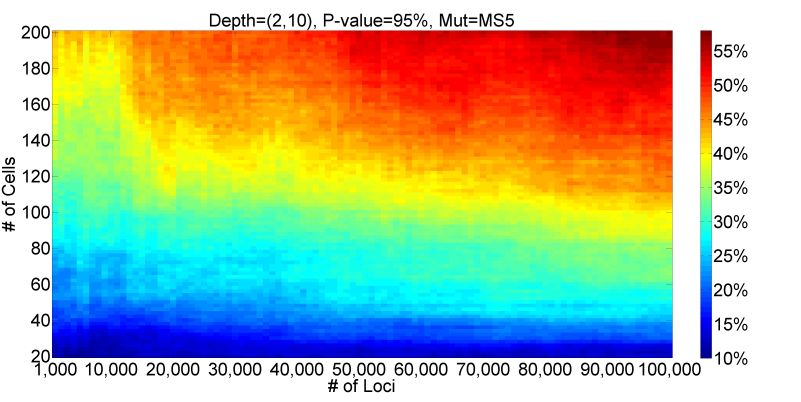

Supplement: S2 File — Results include figures similar to Figs 1B, 2B and 3B but using STR mutations with mutation rates 10−3 and 10−5 and SNV mutations with mutation rates 10−7, 10−8, 10−9 using current and improved values for ADO and noise corresponding to future quality enhancements of single cell genomics. (ZIP) [file pcbi.1004983.s002.zip › Clusters/Clusters_Param_2_10_pval_95_MS5_Res.jpg]

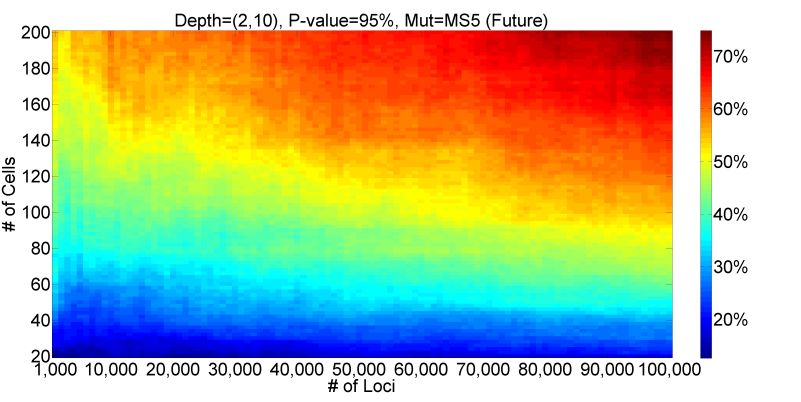

Supplement: S2 File — Results include figures similar to Figs 1B, 2B and 3B but using STR mutations with mutation rates 10−3 and 10−5 and SNV mutations with mutation rates 10−7, 10−8, 10−9 using current and improved values for ADO and noise corresponding to future quality enhancements of single cell genomics. (ZIP) [file pcbi.1004983.s002.zip › Clusters/Clusters_Param_2_10_pval_95_MS5_Res_Future.jpg]

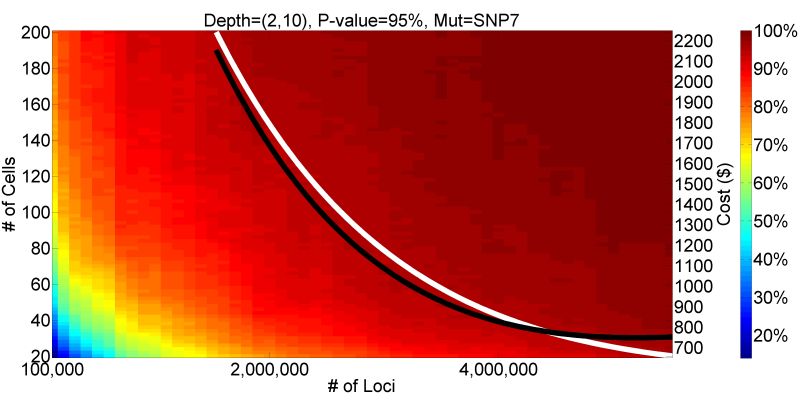

Supplement: S2 File — Results include figures similar to Figs 1B, 2B and 3B but using STR mutations with mutation rates 10−3 and 10−5 and SNV mutations with mutation rates 10−7, 10−8, 10−9 using current and improved values for ADO and noise corresponding to future quality enhancements of single cell genomics. (ZIP) [file pcbi.1004983.s002.zip › Clusters/Clusters_Param_2_10_pval_95_SNP7_Res.jpg]

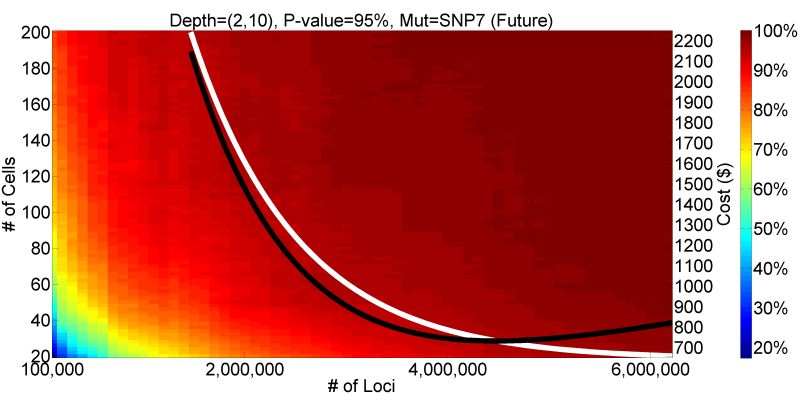

Supplement: S2 File — Results include figures similar to Figs 1B, 2B and 3B but using STR mutations with mutation rates 10−3 and 10−5 and SNV mutations with mutation rates 10−7, 10−8, 10−9 using current and improved values for ADO and noise corresponding to future quality enhancements of single cell genomics. (ZIP) [file pcbi.1004983.s002.zip › Clusters/Clusters_Param_2_10_pval_95_SNP7_Res_Future.jpg]

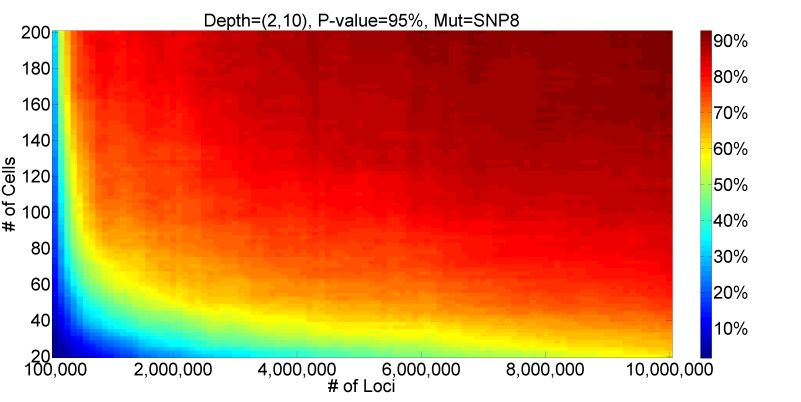

Supplement: S2 File — Results include figures similar to Figs 1B, 2B and 3B but using STR mutations with mutation rates 10−3 and 10−5 and SNV mutations with mutation rates 10−7, 10−8, 10−9 using current and improved values for ADO and noise corresponding to future quality enhancements of single cell genomics. (ZIP) [file pcbi.1004983.s002.zip › Clusters/Clusters_Param_2_10_pval_95_SNP8_Res.jpg]

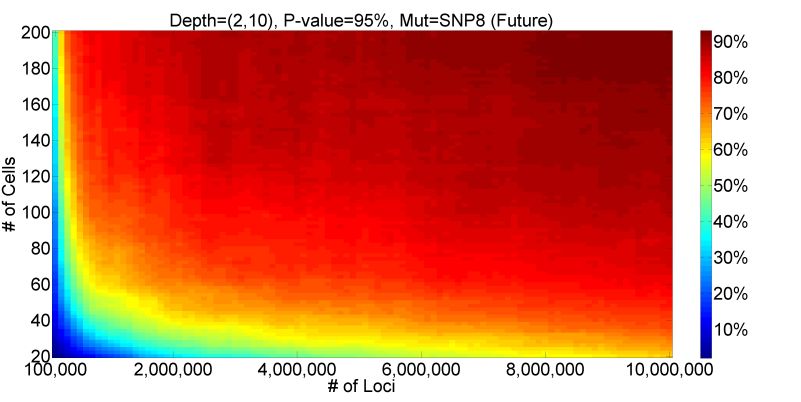

Supplement: S2 File — Results include figures similar to Figs 1B, 2B and 3B but using STR mutations with mutation rates 10−3 and 10−5 and SNV mutations with mutation rates 10−7, 10−8, 10−9 using current and improved values for ADO and noise corresponding to future quality enhancements of single cell genomics. (ZIP) [file pcbi.1004983.s002.zip › Clusters/Clusters_Param_2_10_pval_95_SNP8_Res_Future.jpg]

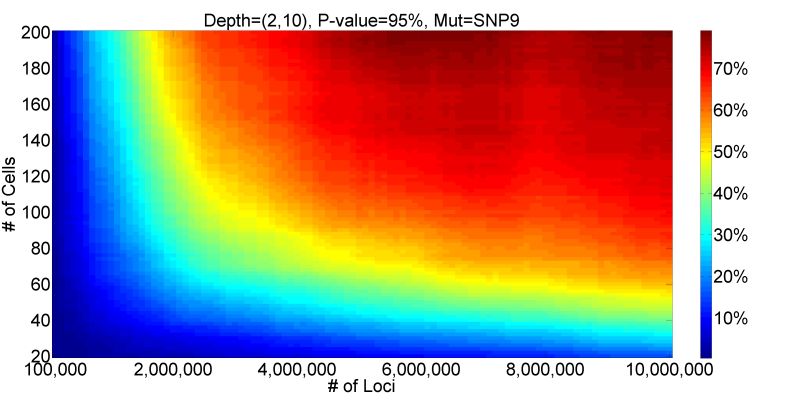

Supplement: S2 File — Results include figures similar to Figs 1B, 2B and 3B but using STR mutations with mutation rates 10−3 and 10−5 and SNV mutations with mutation rates 10−7, 10−8, 10−9 using current and improved values for ADO and noise corresponding to future quality enhancements of single cell genomics. (ZIP) [file pcbi.1004983.s002.zip › Clusters/Clusters_Param_2_10_pval_95_SNP9_Res.jpg]

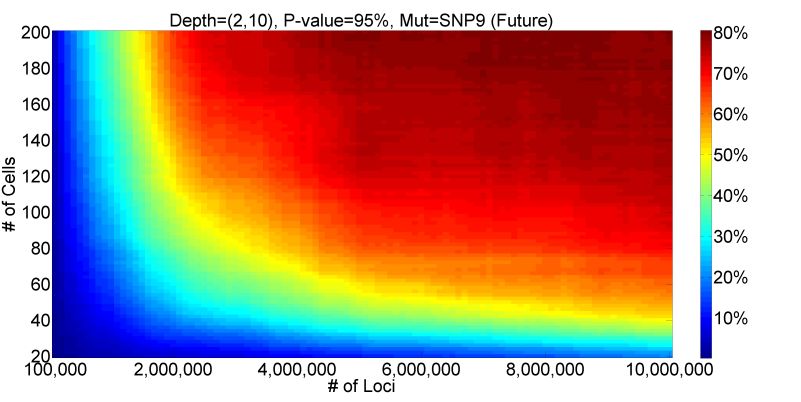

Supplement: S2 File — Results include figures similar to Figs 1B, 2B and 3B but using STR mutations with mutation rates 10−3 and 10−5 and SNV mutations with mutation rates 10−7, 10−8, 10−9 using current and improved values for ADO and noise corresponding to future quality enhancements of single cell genomics. (ZIP) [file pcbi.1004983.s002.zip › Clusters/Clusters_Param_2_10_pval_95_SNP9_Res_Future.jpg]

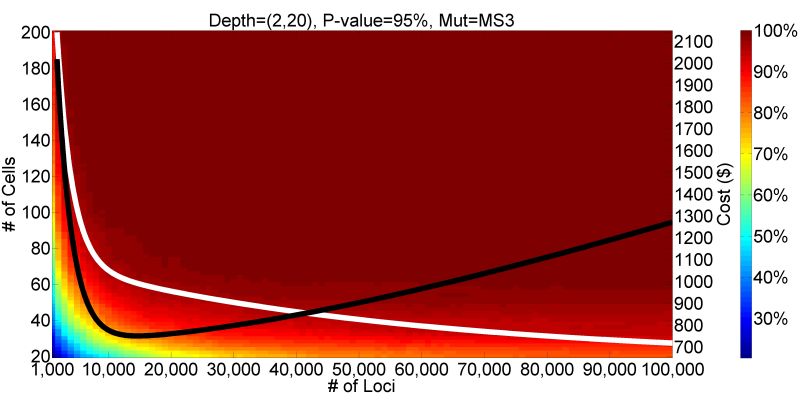

Supplement: S2 File — Results include figures similar to Figs 1B, 2B and 3B but using STR mutations with mutation rates 10−3 and 10−5 and SNV mutations with mutation rates 10−7, 10−8, 10−9 using current and improved values for ADO and noise corresponding to future quality enhancements of single cell genomics. (ZIP) [file pcbi.1004983.s002.zip › Clusters/Clusters_Param_2_20_pval_95_MS3_Res.jpg]

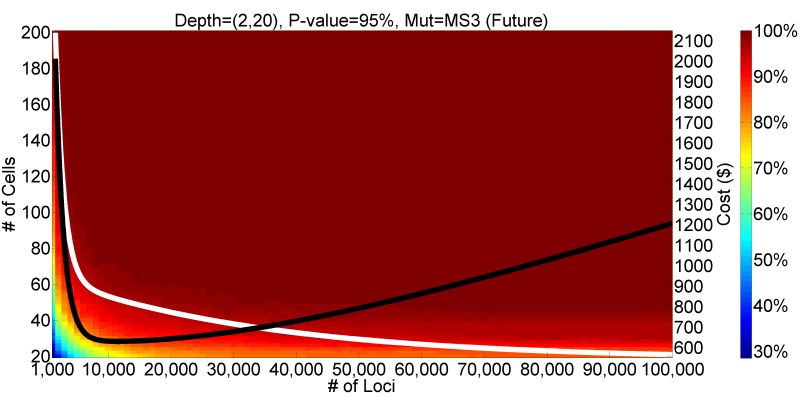

Supplement: S2 File — Results include figures similar to Figs 1B, 2B and 3B but using STR mutations with mutation rates 10−3 and 10−5 and SNV mutations with mutation rates 10−7, 10−8, 10−9 using current and improved values for ADO and noise corresponding to future quality enhancements of single cell genomics. (ZIP) [file pcbi.1004983.s002.zip › Clusters/Clusters_Param_2_20_pval_95_MS3_Res_Future.jpg]

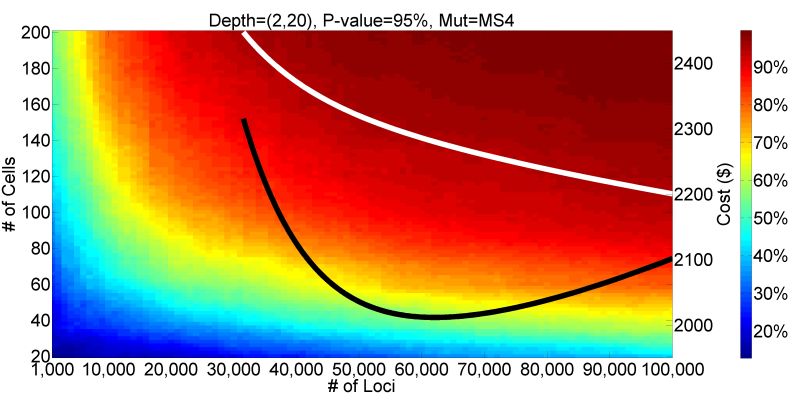

Supplement: S2 File — Results include figures similar to Figs 1B, 2B and 3B but using STR mutations with mutation rates 10−3 and 10−5 and SNV mutations with mutation rates 10−7, 10−8, 10−9 using current and improved values for ADO and noise corresponding to future quality enhancements of single cell genomics. (ZIP) [file pcbi.1004983.s002.zip › Clusters/Clusters_Param_2_20_pval_95_MS4_Res.jpg]

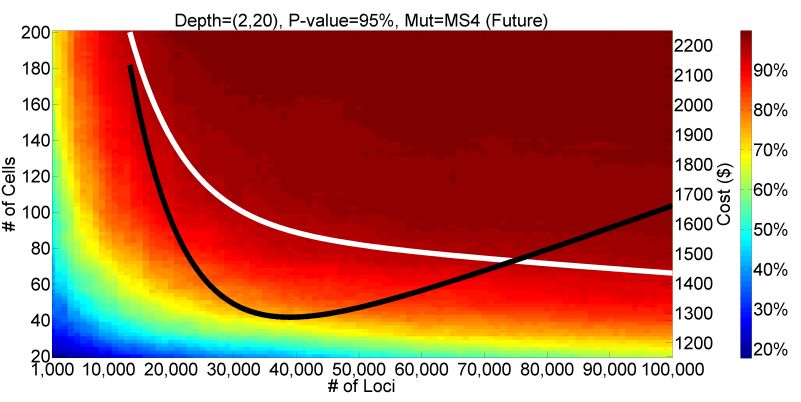

Supplement: S2 File — Results include figures similar to Figs 1B, 2B and 3B but using STR mutations with mutation rates 10−3 and 10−5 and SNV mutations with mutation rates 10−7, 10−8, 10−9 using current and improved values for ADO and noise corresponding to future quality enhancements of single cell genomics. (ZIP) [file pcbi.1004983.s002.zip › Clusters/Clusters_Param_2_20_pval_95_MS4_Res_Future.jpg]

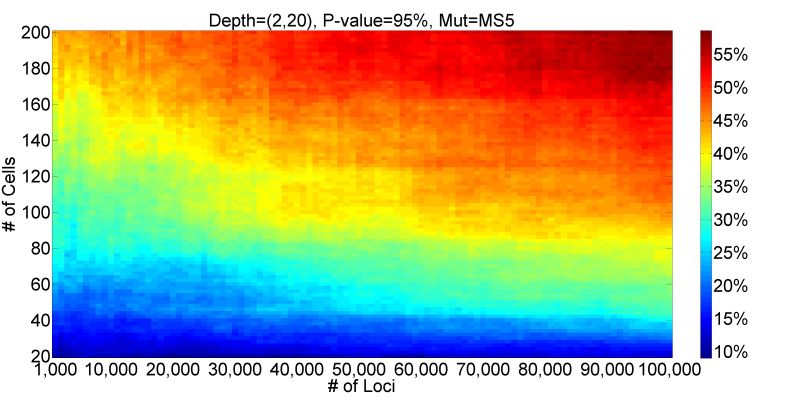

Supplement: S2 File — Results include figures similar to Figs 1B, 2B and 3B but using STR mutations with mutation rates 10−3 and 10−5 and SNV mutations with mutation rates 10−7, 10−8, 10−9 using current and improved values for ADO and noise corresponding to future quality enhancements of single cell genomics. (ZIP) [file pcbi.1004983.s002.zip › Clusters/Clusters_Param_2_20_pval_95_MS5_Res.jpg]

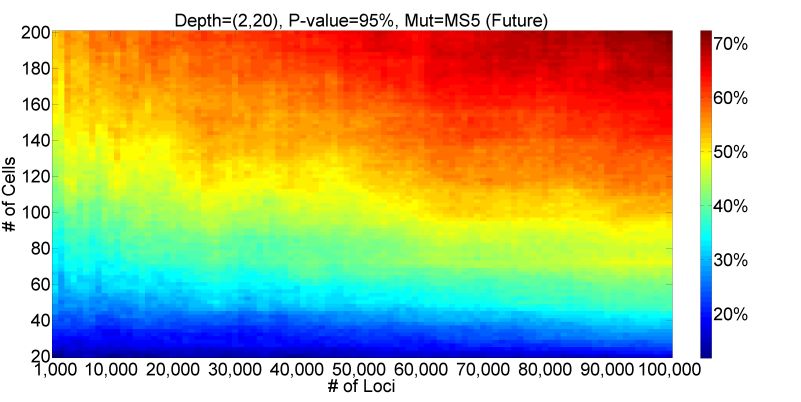

Supplement: S2 File — Results include figures similar to Figs 1B, 2B and 3B but using STR mutations with mutation rates 10−3 and 10−5 and SNV mutations with mutation rates 10−7, 10−8, 10−9 using current and improved values for ADO and noise corresponding to future quality enhancements of single cell genomics. (ZIP) [file pcbi.1004983.s002.zip › Clusters/Clusters_Param_2_20_pval_95_MS5_Res_Future.jpg]

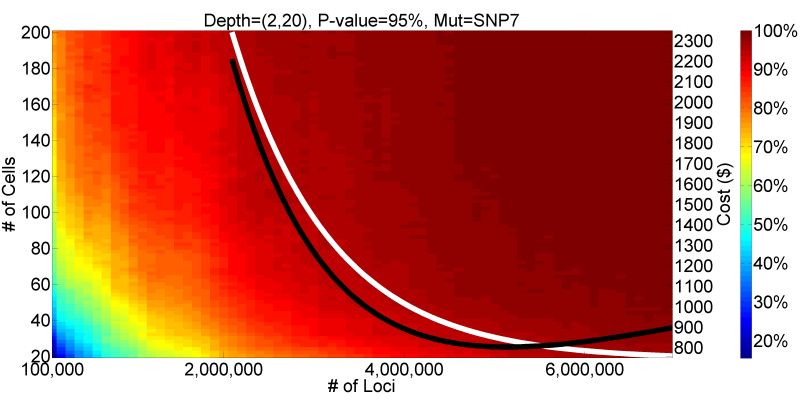

Supplement: S2 File — Results include figures similar to Figs 1B, 2B and 3B but using STR mutations with mutation rates 10−3 and 10−5 and SNV mutations with mutation rates 10−7, 10−8, 10−9 using current and improved values for ADO and noise corresponding to future quality enhancements of single cell genomics. (ZIP) [file pcbi.1004983.s002.zip › Clusters/Clusters_Param_2_20_pval_95_SNP7_Res.jpg]

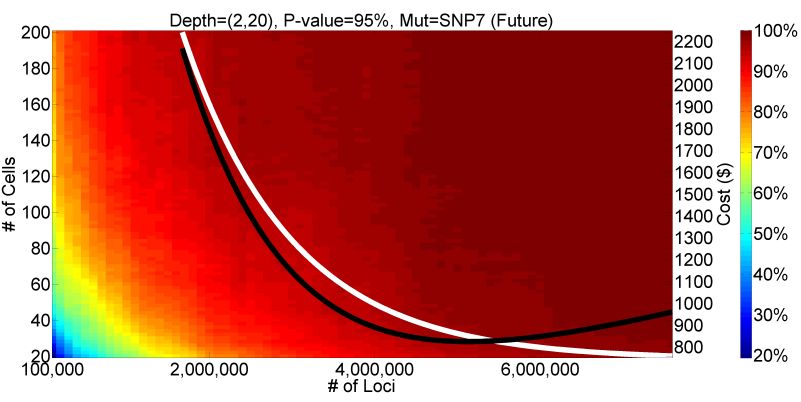

Supplement: S2 File — Results include figures similar to Figs 1B, 2B and 3B but using STR mutations with mutation rates 10−3 and 10−5 and SNV mutations with mutation rates 10−7, 10−8, 10−9 using current and improved values for ADO and noise corresponding to future quality enhancements of single cell genomics. (ZIP) [file pcbi.1004983.s002.zip › Clusters/Clusters_Param_2_20_pval_95_SNP7_Res_Future.jpg]

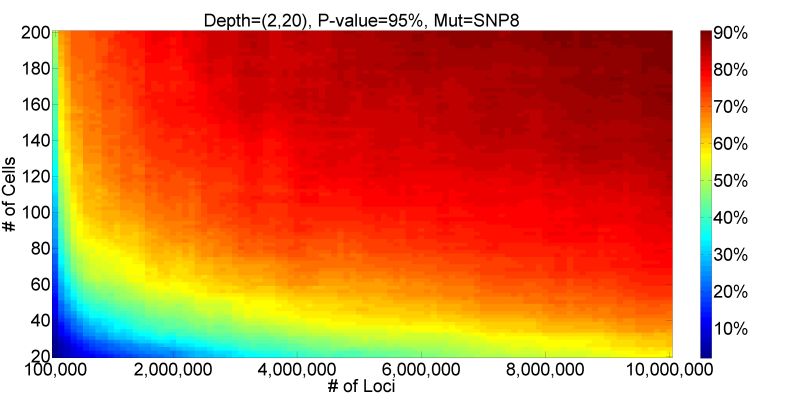

Supplement: S2 File — Results include figures similar to Figs 1B, 2B and 3B but using STR mutations with mutation rates 10−3 and 10−5 and SNV mutations with mutation rates 10−7, 10−8, 10−9 using current and improved values for ADO and noise corresponding to future quality enhancements of single cell genomics. (ZIP) [file pcbi.1004983.s002.zip › Clusters/Clusters_Param_2_20_pval_95_SNP8_Res.jpg]

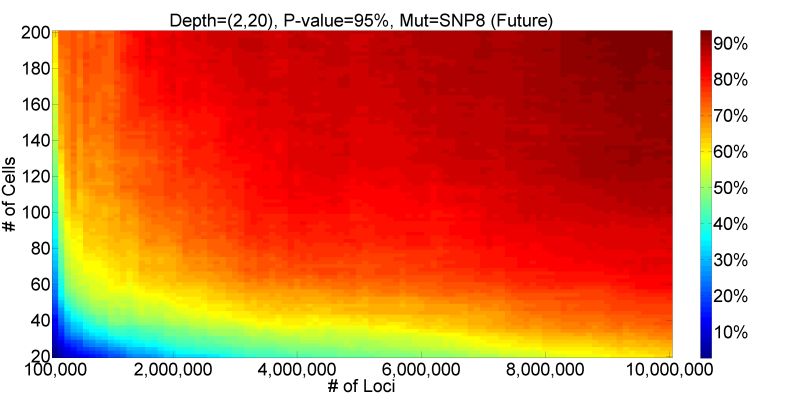

Supplement: S2 File — Results include figures similar to Figs 1B, 2B and 3B but using STR mutations with mutation rates 10−3 and 10−5 and SNV mutations with mutation rates 10−7, 10−8, 10−9 using current and improved values for ADO and noise corresponding to future quality enhancements of single cell genomics. (ZIP) [file pcbi.1004983.s002.zip › Clusters/Clusters_Param_2_20_pval_95_SNP8_Res_Future.jpg]

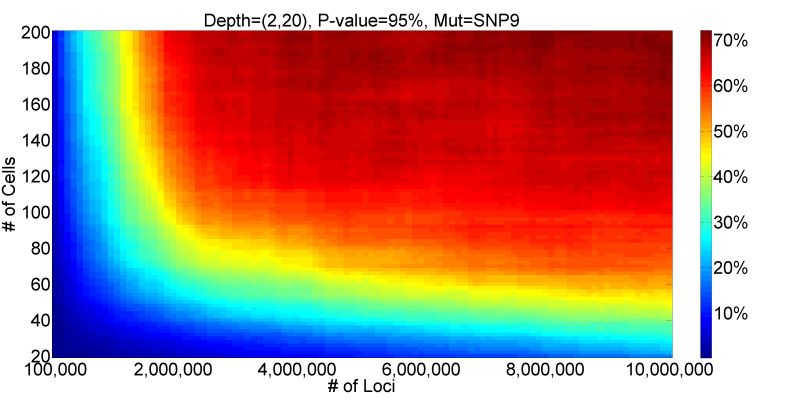

Supplement: S2 File — Results include figures similar to Figs 1B, 2B and 3B but using STR mutations with mutation rates 10−3 and 10−5 and SNV mutations with mutation rates 10−7, 10−8, 10−9 using current and improved values for ADO and noise corresponding to future quality enhancements of single cell genomics. (ZIP) [file pcbi.1004983.s002.zip › Clusters/Clusters_Param_2_20_pval_95_SNP9_Res.jpg]

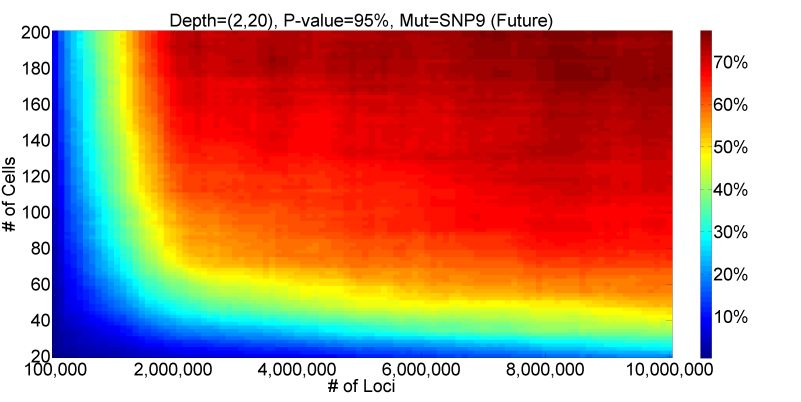

Supplement: S2 File — Results include figures similar to Figs 1B, 2B and 3B but using STR mutations with mutation rates 10−3 and 10−5 and SNV mutations with mutation rates 10−7, 10−8, 10−9 using current and improved values for ADO and noise corresponding to future quality enhancements of single cell genomics. (ZIP) [file pcbi.1004983.s002.zip › Clusters/Clusters_Param_2_20_pval_95_SNP9_Res_Future.jpg]

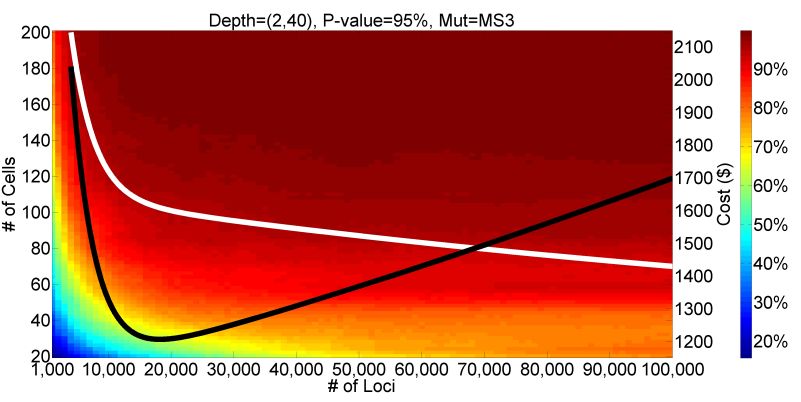

Supplement: S2 File — Results include figures similar to Figs 1B, 2B and 3B but using STR mutations with mutation rates 10−3 and 10−5 and SNV mutations with mutation rates 10−7, 10−8, 10−9 using current and improved values for ADO and noise corresponding to future quality enhancements of single cell genomics. (ZIP) [file pcbi.1004983.s002.zip › Clusters/Clusters_Param_2_40_pval_95_MS3_Res.jpg]

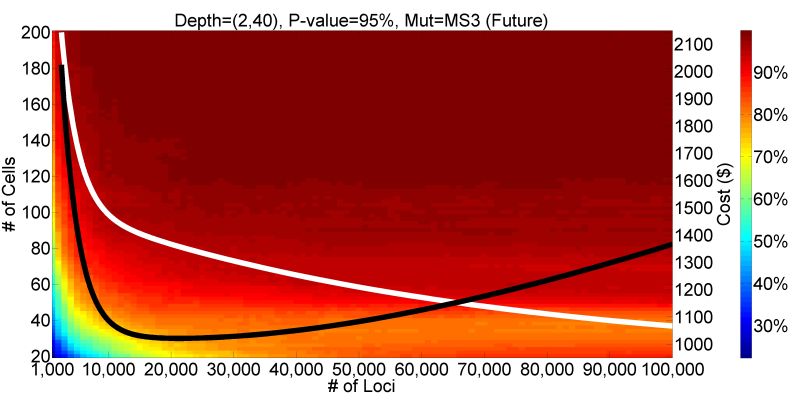

Supplement: S2 File — Results include figures similar to Figs 1B, 2B and 3B but using STR mutations with mutation rates 10−3 and 10−5 and SNV mutations with mutation rates 10−7, 10−8, 10−9 using current and improved values for ADO and noise corresponding to future quality enhancements of single cell genomics. (ZIP) [file pcbi.1004983.s002.zip › Clusters/Clusters_Param_2_40_pval_95_MS3_Res_Future.jpg]

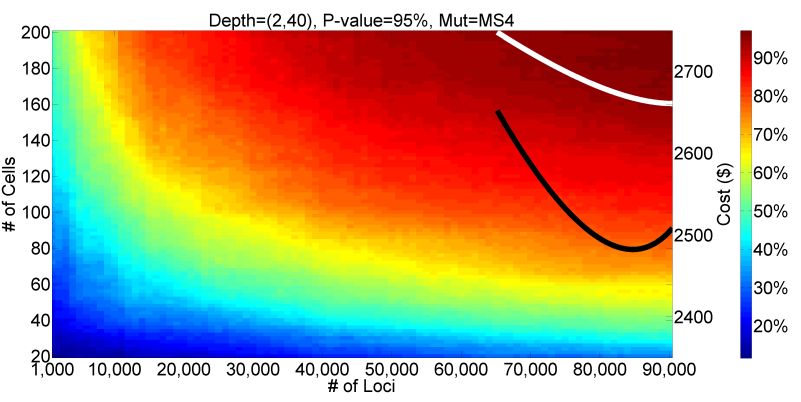

Supplement: S2 File — Results include figures similar to Figs 1B, 2B and 3B but using STR mutations with mutation rates 10−3 and 10−5 and SNV mutations with mutation rates 10−7, 10−8, 10−9 using current and improved values for ADO and noise corresponding to future quality enhancements of single cell genomics. (ZIP) [file pcbi.1004983.s002.zip › Clusters/Clusters_Param_2_40_pval_95_MS4_Res.jpg]

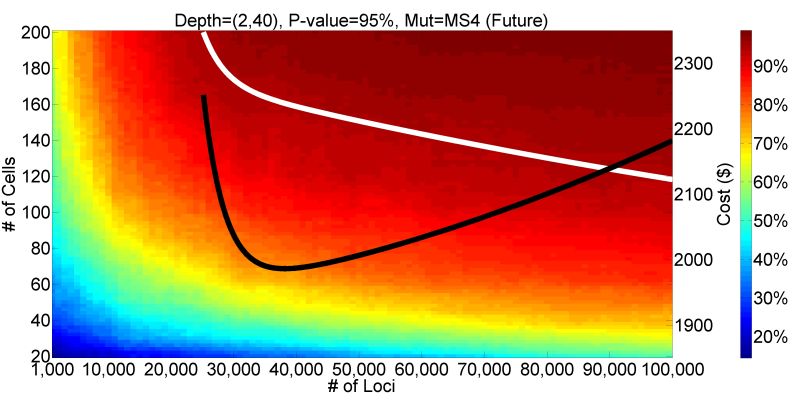

Supplement: S2 File — Results include figures similar to Figs 1B, 2B and 3B but using STR mutations with mutation rates 10−3 and 10−5 and SNV mutations with mutation rates 10−7, 10−8, 10−9 using current and improved values for ADO and noise corresponding to future quality enhancements of single cell genomics. (ZIP) [file pcbi.1004983.s002.zip › Clusters/Clusters_Param_2_40_pval_95_MS4_Res_Future.jpg]

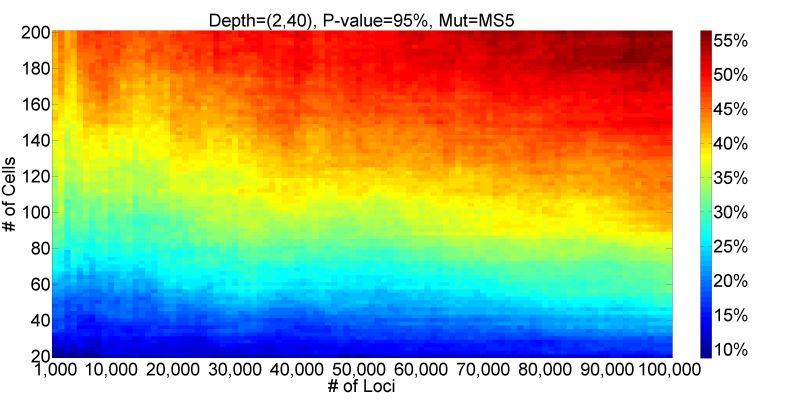

Supplement: S2 File — Results include figures similar to Figs 1B, 2B and 3B but using STR mutations with mutation rates 10−3 and 10−5 and SNV mutations with mutation rates 10−7, 10−8, 10−9 using current and improved values for ADO and noise corresponding to future quality enhancements of single cell genomics. (ZIP) [file pcbi.1004983.s002.zip › Clusters/Clusters_Param_2_40_pval_95_MS5_Res.jpg]

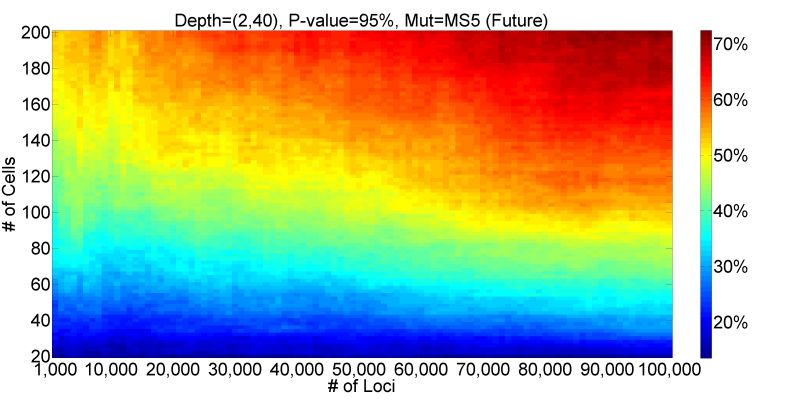

Supplement: S2 File — Results include figures similar to Figs 1B, 2B and 3B but using STR mutations with mutation rates 10−3 and 10−5 and SNV mutations with mutation rates 10−7, 10−8, 10−9 using current and improved values for ADO and noise corresponding to future quality enhancements of single cell genomics. (ZIP) [file pcbi.1004983.s002.zip › Clusters/Clusters_Param_2_40_pval_95_MS5_Res_Future.jpg]

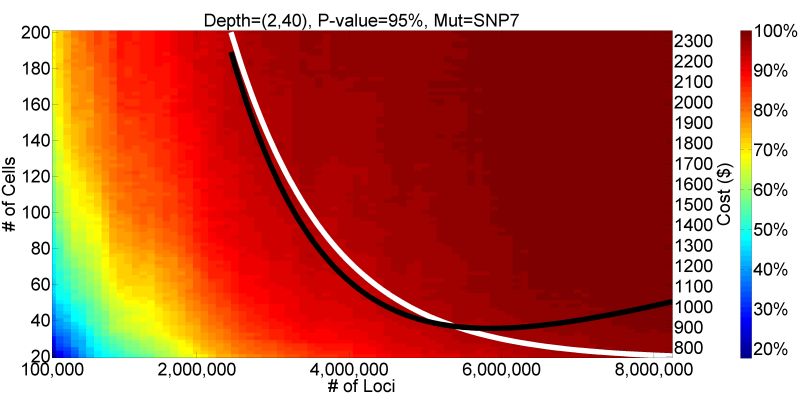

Supplement: S2 File — Results include figures similar to Figs 1B, 2B and 3B but using STR mutations with mutation rates 10−3 and 10−5 and SNV mutations with mutation rates 10−7, 10−8, 10−9 using current and improved values for ADO and noise corresponding to future quality enhancements of single cell genomics. (ZIP) [file pcbi.1004983.s002.zip › Clusters/Clusters_Param_2_40_pval_95_SNP7_Res.jpg]

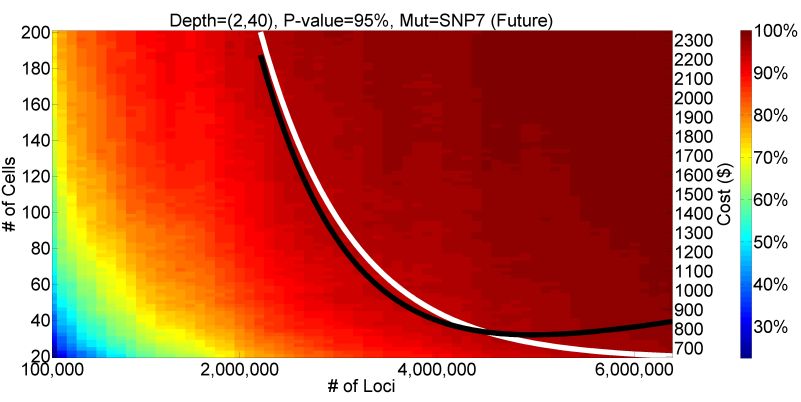

Supplement: S2 File — Results include figures similar to Figs 1B, 2B and 3B but using STR mutations with mutation rates 10−3 and 10−5 and SNV mutations with mutation rates 10−7, 10−8, 10−9 using current and improved values for ADO and noise corresponding to future quality enhancements of single cell genomics. (ZIP) [file pcbi.1004983.s002.zip › Clusters/Clusters_Param_2_40_pval_95_SNP7_Res_Future.jpg]

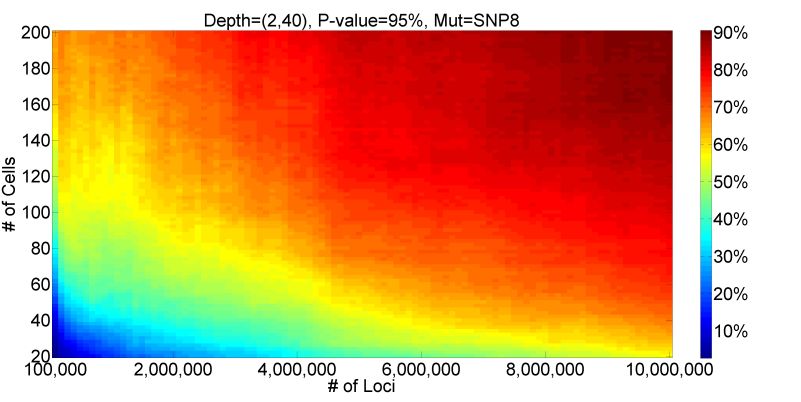

Supplement: S2 File — Results include figures similar to Figs 1B, 2B and 3B but using STR mutations with mutation rates 10−3 and 10−5 and SNV mutations with mutation rates 10−7, 10−8, 10−9 using current and improved values for ADO and noise corresponding to future quality enhancements of single cell genomics. (ZIP) [file pcbi.1004983.s002.zip › Clusters/Clusters_Param_2_40_pval_95_SNP8_Res.jpg]

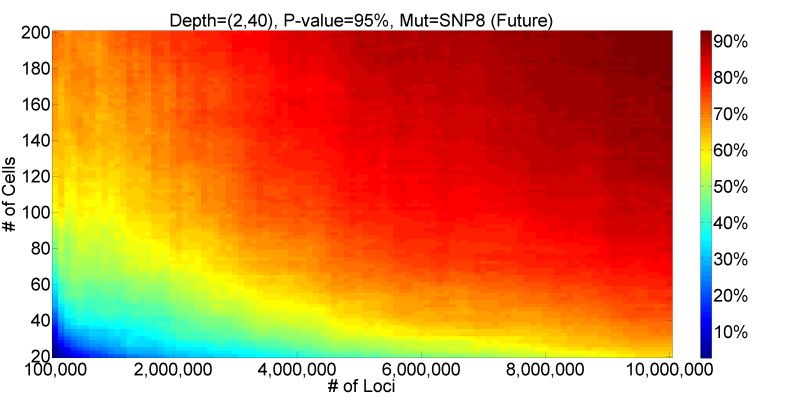

Supplement: S2 File — Results include figures similar to Figs 1B, 2B and 3B but using STR mutations with mutation rates 10−3 and 10−5 and SNV mutations with mutation rates 10−7, 10−8, 10−9 using current and improved values for ADO and noise corresponding to future quality enhancements of single cell genomics. (ZIP) [file pcbi.1004983.s002.zip › Clusters/Clusters_Param_2_40_pval_95_SNP8_Res_Future.jpg]

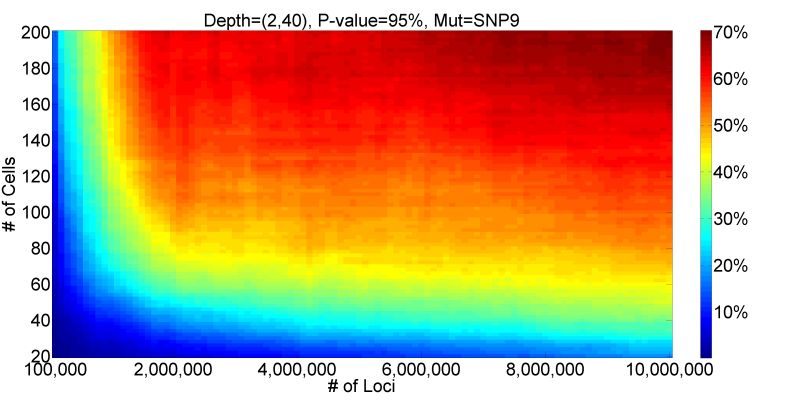

Supplement: S2 File — Results include figures similar to Figs 1B, 2B and 3B but using STR mutations with mutation rates 10−3 and 10−5 and SNV mutations with mutation rates 10−7, 10−8, 10−9 using current and improved values for ADO and noise corresponding to future quality enhancements of single cell genomics. (ZIP) [file pcbi.1004983.s002.zip › Clusters/Clusters_Param_2_40_pval_95_SNP9_Res.jpg]

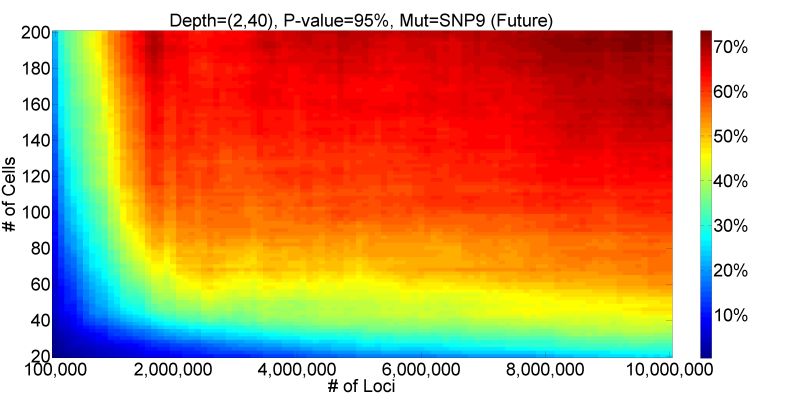

Supplement: S2 File — Results include figures similar to Figs 1B, 2B and 3B but using STR mutations with mutation rates 10−3 and 10−5 and SNV mutations with mutation rates 10−7, 10−8, 10−9 using current and improved values for ADO and noise corresponding to future quality enhancements of single cell genomics. (ZIP) [file pcbi.1004983.s002.zip › Clusters/Clusters_Param_2_40_pval_95_SNP9_Res_Future.jpg]

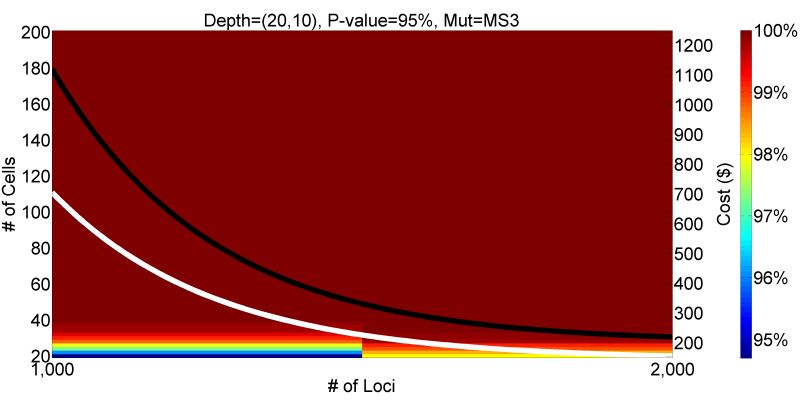

Supplement: S2 File — Results include figures similar to Figs 1B, 2B and 3B but using STR mutations with mutation rates 10−3 and 10−5 and SNV mutations with mutation rates 10−7, 10−8, 10−9 using current and improved values for ADO and noise corresponding to future quality enhancements of single cell genomics. (ZIP) [file pcbi.1004983.s002.zip › Clusters/Clusters_Param_20_10_pval_95_MS3_Res.jpg]

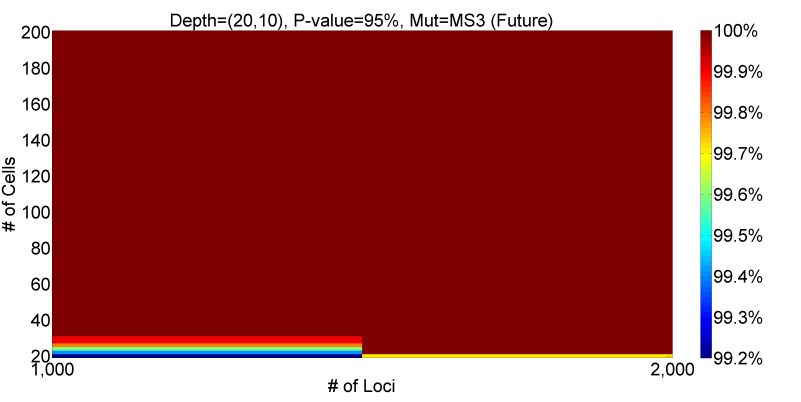

Supplement: S2 File — Results include figures similar to Figs 1B, 2B and 3B but using STR mutations with mutation rates 10−3 and 10−5 and SNV mutations with mutation rates 10−7, 10−8, 10−9 using current and improved values for ADO and noise corresponding to future quality enhancements of single cell genomics. (ZIP) [file pcbi.1004983.s002.zip › Clusters/Clusters_Param_20_10_pval_95_MS3_Res_Future.jpg]

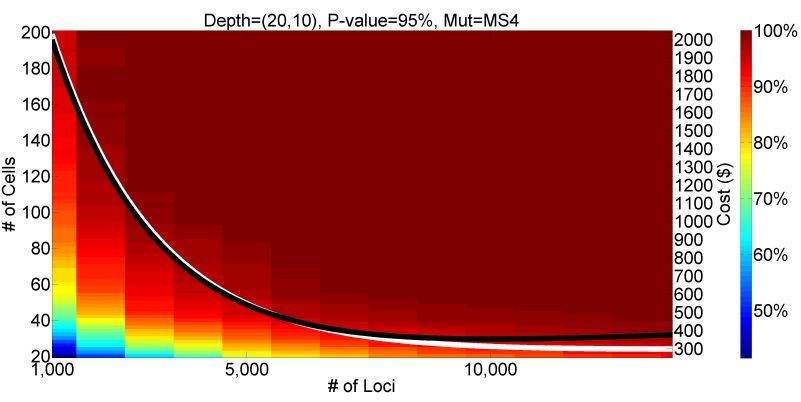

Supplement: S2 File — Results include figures similar to Figs 1B, 2B and 3B but using STR mutations with mutation rates 10−3 and 10−5 and SNV mutations with mutation rates 10−7, 10−8, 10−9 using current and improved values for ADO and noise corresponding to future quality enhancements of single cell genomics. (ZIP) [file pcbi.1004983.s002.zip › Clusters/Clusters_Param_20_10_pval_95_MS4_Res.jpg]

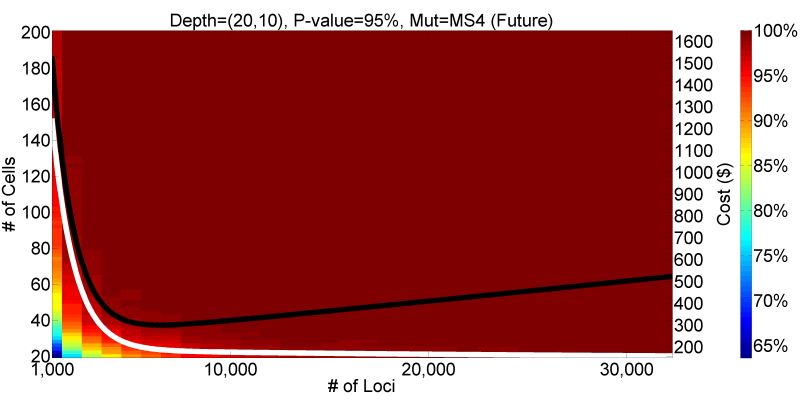

Supplement: S2 File — Results include figures similar to Figs 1B, 2B and 3B but using STR mutations with mutation rates 10−3 and 10−5 and SNV mutations with mutation rates 10−7, 10−8, 10−9 using current and improved values for ADO and noise corresponding to future quality enhancements of single cell genomics. (ZIP) [file pcbi.1004983.s002.zip › Clusters/Clusters_Param_20_10_pval_95_MS4_Res_Future.jpg]

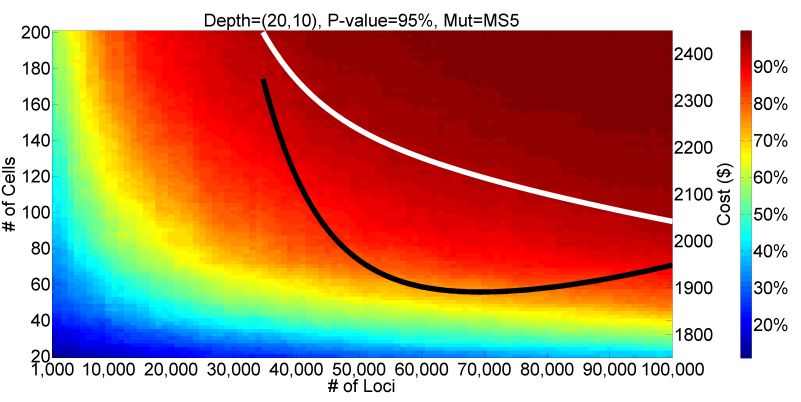

Supplement: S2 File — Results include figures similar to Figs 1B, 2B and 3B but using STR mutations with mutation rates 10−3 and 10−5 and SNV mutations with mutation rates 10−7, 10−8, 10−9 using current and improved values for ADO and noise corresponding to future quality enhancements of single cell genomics. (ZIP) [file pcbi.1004983.s002.zip › Clusters/Clusters_Param_20_10_pval_95_MS5_Res.jpg]

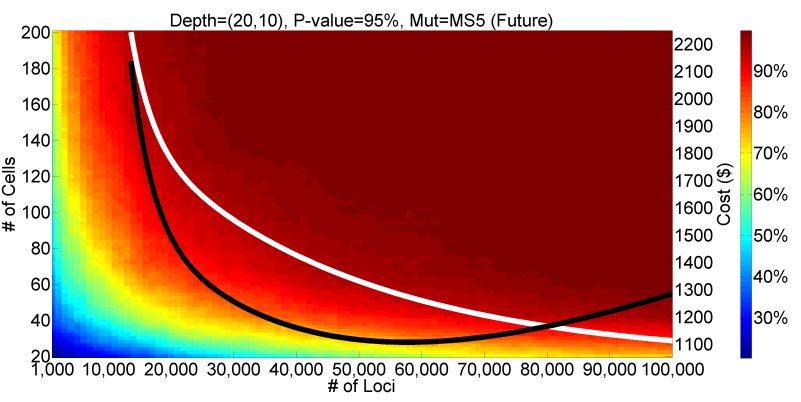

Supplement: S2 File — Results include figures similar to Figs 1B, 2B and 3B but using STR mutations with mutation rates 10−3 and 10−5 and SNV mutations with mutation rates 10−7, 10−8, 10−9 using current and improved values for ADO and noise corresponding to future quality enhancements of single cell genomics. (ZIP) [file pcbi.1004983.s002.zip › Clusters/Clusters_Param_20_10_pval_95_MS5_Res_Future.jpg]

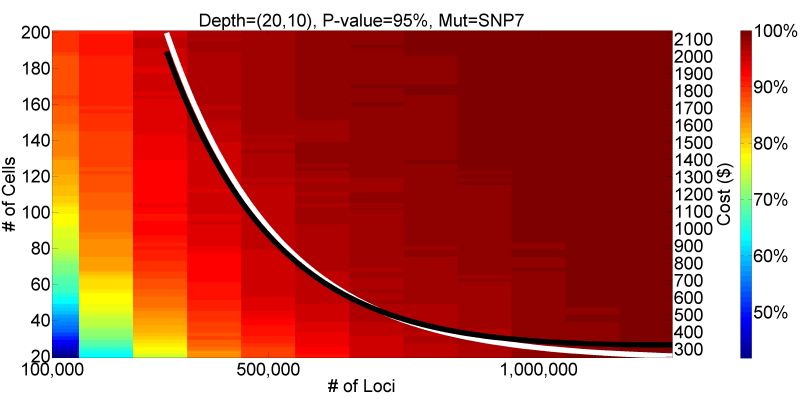

Supplement: S2 File — Results include figures similar to Figs 1B, 2B and 3B but using STR mutations with mutation rates 10−3 and 10−5 and SNV mutations with mutation rates 10−7, 10−8, 10−9 using current and improved values for ADO and noise corresponding to future quality enhancements of single cell genomics. (ZIP) [file pcbi.1004983.s002.zip › Clusters/Clusters_Param_20_10_pval_95_SNP7_Res.jpg]

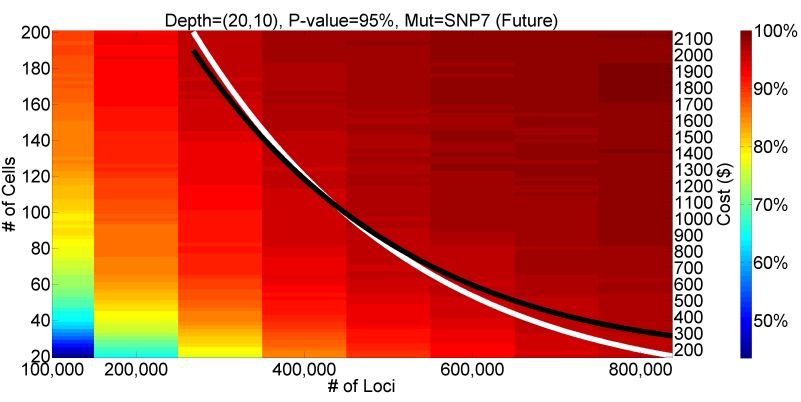

Supplement: S2 File — Results include figures similar to Figs 1B, 2B and 3B but using STR mutations with mutation rates 10−3 and 10−5 and SNV mutations with mutation rates 10−7, 10−8, 10−9 using current and improved values for ADO and noise corresponding to future quality enhancements of single cell genomics. (ZIP) [file pcbi.1004983.s002.zip › Clusters/Clusters_Param_20_10_pval_95_SNP7_Res_Future.jpg]

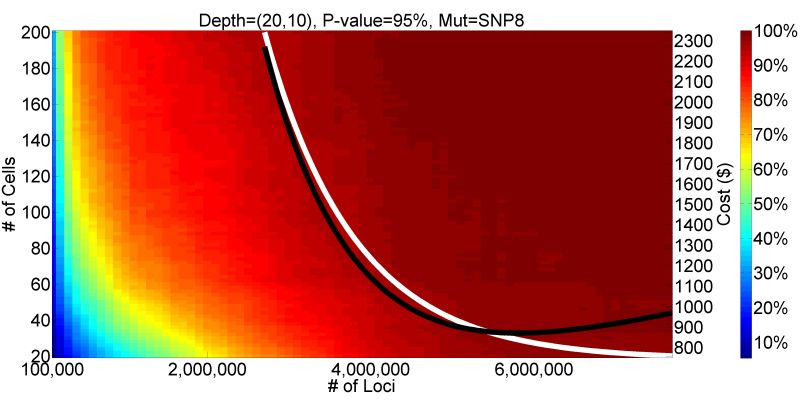

Supplement: S2 File — Results include figures similar to Figs 1B, 2B and 3B but using STR mutations with mutation rates 10−3 and 10−5 and SNV mutations with mutation rates 10−7, 10−8, 10−9 using current and improved values for ADO and noise corresponding to future quality enhancements of single cell genomics. (ZIP) [file pcbi.1004983.s002.zip › Clusters/Clusters_Param_20_10_pval_95_SNP8_Res.jpg]

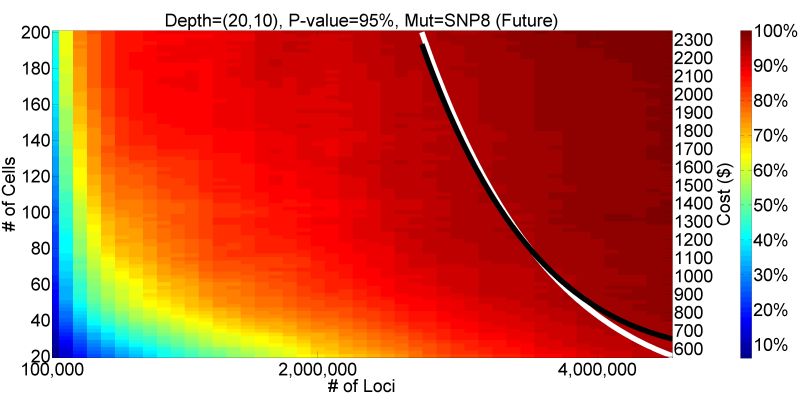

Supplement: S2 File — Results include figures similar to Figs 1B, 2B and 3B but using STR mutations with mutation rates 10−3 and 10−5 and SNV mutations with mutation rates 10−7, 10−8, 10−9 using current and improved values for ADO and noise corresponding to future quality enhancements of single cell genomics. (ZIP) [file pcbi.1004983.s002.zip › Clusters/Clusters_Param_20_10_pval_95_SNP8_Res_Future.jpg]

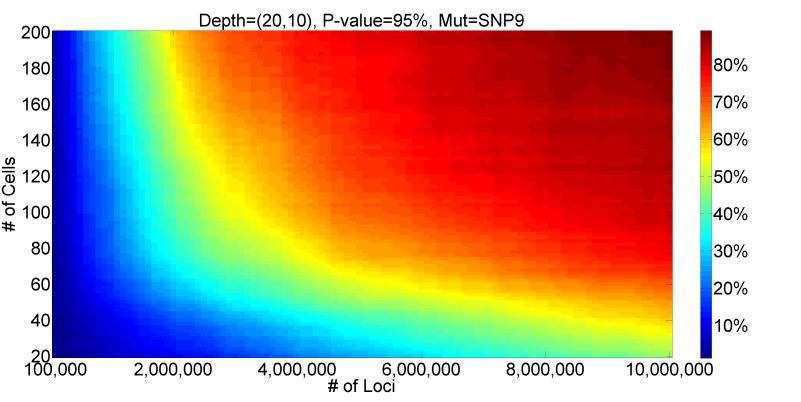

Supplement: S2 File — Results include figures similar to Figs 1B, 2B and 3B but using STR mutations with mutation rates 10−3 and 10−5 and SNV mutations with mutation rates 10−7, 10−8, 10−9 using current and improved values for ADO and noise corresponding to future quality enhancements of single cell genomics. (ZIP) [file pcbi.1004983.s002.zip › Clusters/Clusters_Param_20_10_pval_95_SNP9_Res.jpg]

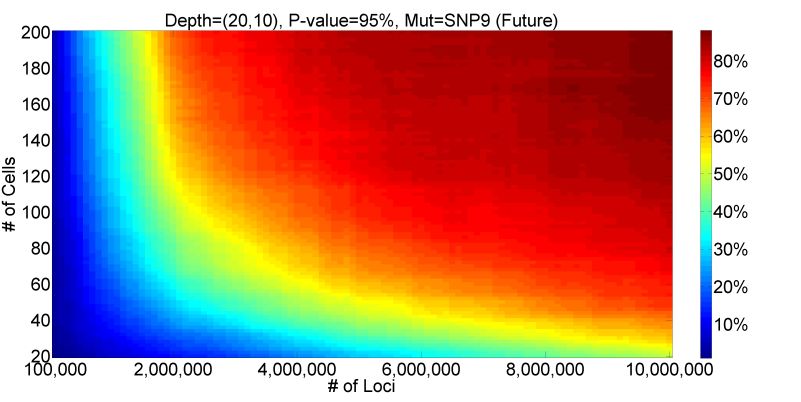

Supplement: S2 File — Results include figures similar to Figs 1B, 2B and 3B but using STR mutations with mutation rates 10−3 and 10−5 and SNV mutations with mutation rates 10−7, 10−8, 10−9 using current and improved values for ADO and noise corresponding to future quality enhancements of single cell genomics. (ZIP) [file pcbi.1004983.s002.zip › Clusters/Clusters_Param_20_10_pval_95_SNP9_Res_Future.jpg]

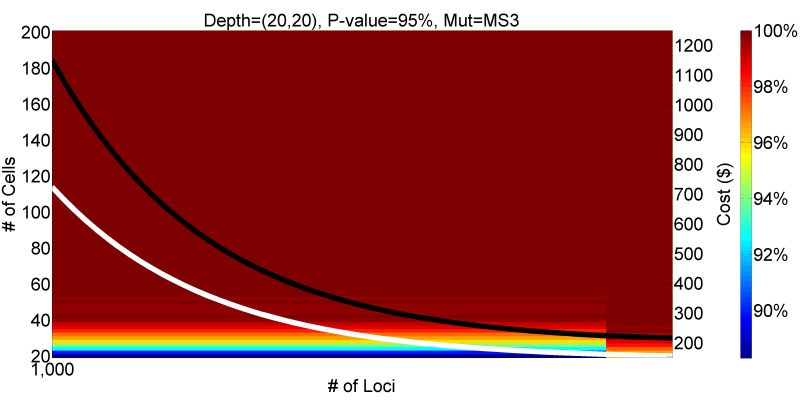

Supplement: S2 File — Results include figures similar to Figs 1B, 2B and 3B but using STR mutations with mutation rates 10−3 and 10−5 and SNV mutations with mutation rates 10−7, 10−8, 10−9 using current and improved values for ADO and noise corresponding to future quality enhancements of single cell genomics. (ZIP) [file pcbi.1004983.s002.zip › Clusters/Clusters_Param_20_20_pval_95_MS3_Res.jpg]

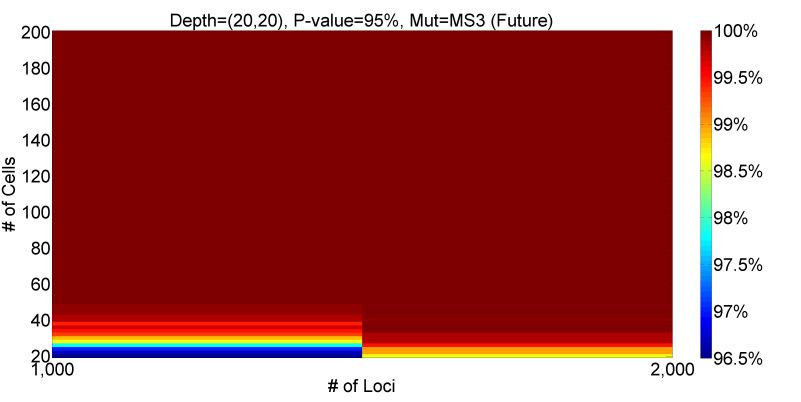

Supplement: S2 File — Results include figures similar to Figs 1B, 2B and 3B but using STR mutations with mutation rates 10−3 and 10−5 and SNV mutations with mutation rates 10−7, 10−8, 10−9 using current and improved values for ADO and noise corresponding to future quality enhancements of single cell genomics. (ZIP) [file pcbi.1004983.s002.zip › Clusters/Clusters_Param_20_20_pval_95_MS3_Res_Future.jpg]

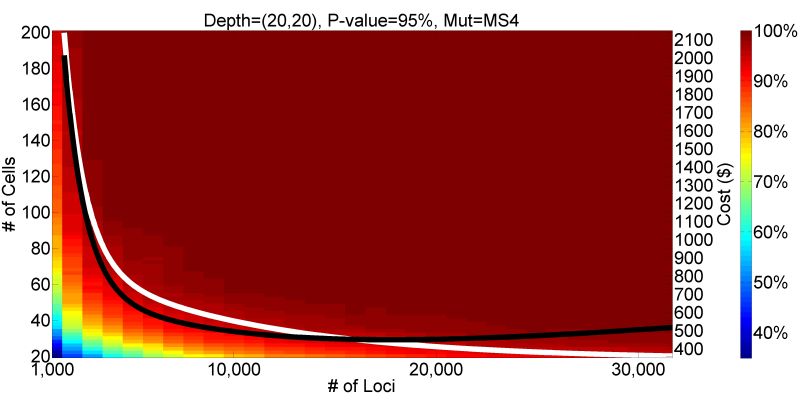

Supplement: S2 File — Results include figures similar to Figs 1B, 2B and 3B but using STR mutations with mutation rates 10−3 and 10−5 and SNV mutations with mutation rates 10−7, 10−8, 10−9 using current and improved values for ADO and noise corresponding to future quality enhancements of single cell genomics. (ZIP) [file pcbi.1004983.s002.zip › Clusters/Clusters_Param_20_20_pval_95_MS4_Res.jpg]

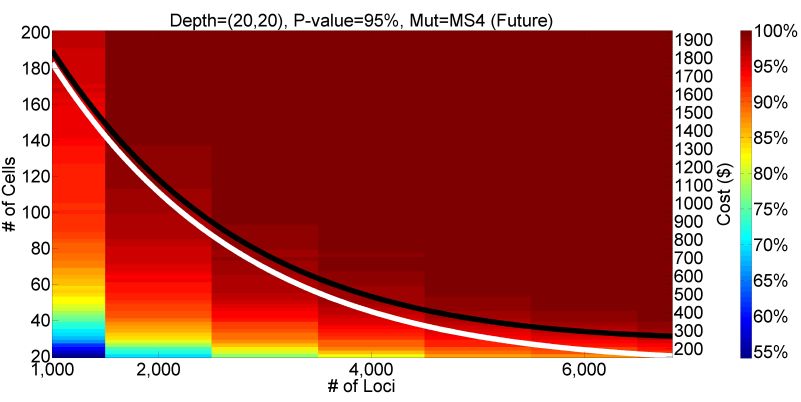

Supplement: S2 File — Results include figures similar to Figs 1B, 2B and 3B but using STR mutations with mutation rates 10−3 and 10−5 and SNV mutations with mutation rates 10−7, 10−8, 10−9 using current and improved values for ADO and noise corresponding to future quality enhancements of single cell genomics. (ZIP) [file pcbi.1004983.s002.zip › Clusters/Clusters_Param_20_20_pval_95_MS4_Res_Future.jpg]

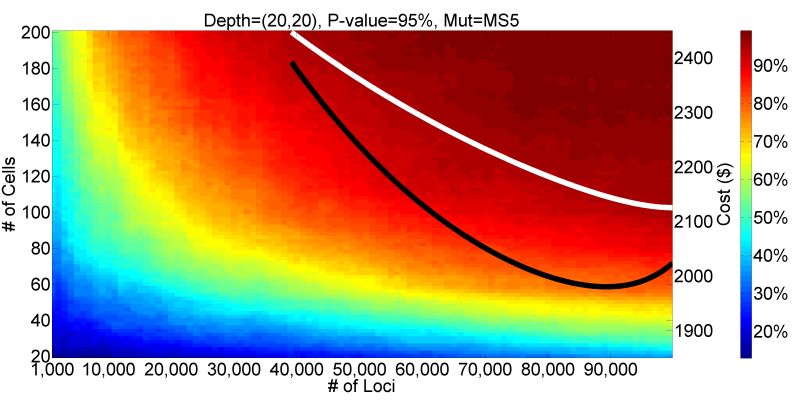

Supplement: S2 File — Results include figures similar to Figs 1B, 2B and 3B but using STR mutations with mutation rates 10−3 and 10−5 and SNV mutations with mutation rates 10−7, 10−8, 10−9 using current and improved values for ADO and noise corresponding to future quality enhancements of single cell genomics. (ZIP) [file pcbi.1004983.s002.zip › Clusters/Clusters_Param_20_20_pval_95_MS5_Res.jpg]

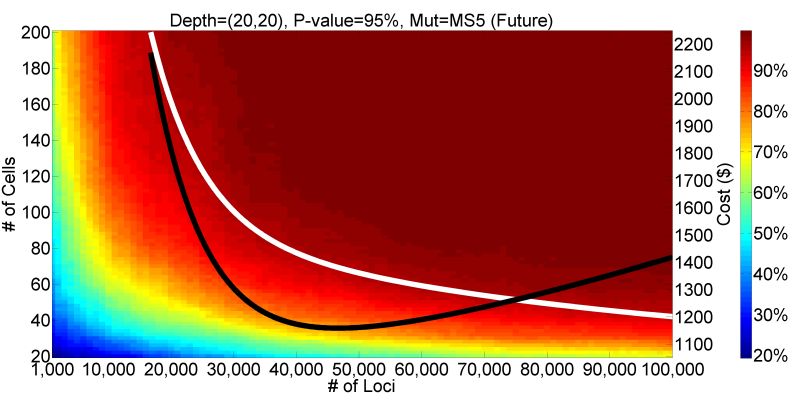

Supplement: S2 File — Results include figures similar to Figs 1B, 2B and 3B but using STR mutations with mutation rates 10−3 and 10−5 and SNV mutations with mutation rates 10−7, 10−8, 10−9 using current and improved values for ADO and noise corresponding to future quality enhancements of single cell genomics. (ZIP) [file pcbi.1004983.s002.zip › Clusters/Clusters_Param_20_20_pval_95_MS5_Res_Future.jpg]

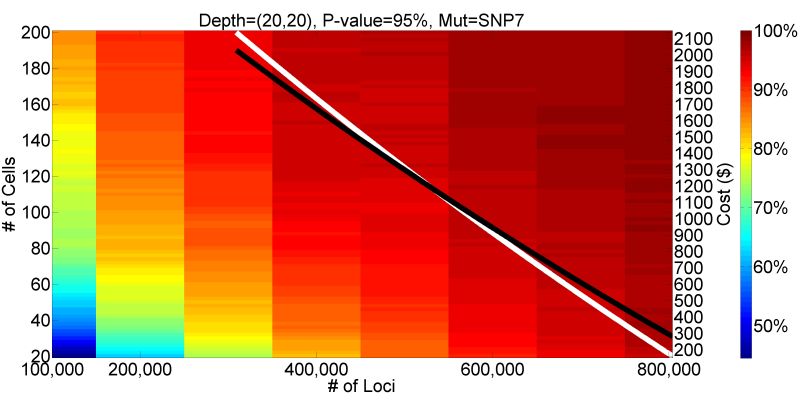

Supplement: S2 File — Results include figures similar to Figs 1B, 2B and 3B but using STR mutations with mutation rates 10−3 and 10−5 and SNV mutations with mutation rates 10−7, 10−8, 10−9 using current and improved values for ADO and noise corresponding to future quality enhancements of single cell genomics. (ZIP) [file pcbi.1004983.s002.zip › Clusters/Clusters_Param_20_20_pval_95_SNP7_Res.jpg]

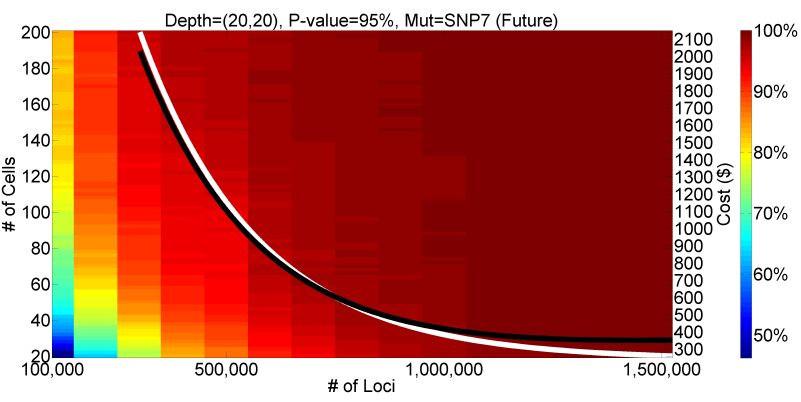

Supplement: S2 File — Results include figures similar to Figs 1B, 2B and 3B but using STR mutations with mutation rates 10−3 and 10−5 and SNV mutations with mutation rates 10−7, 10−8, 10−9 using current and improved values for ADO and noise corresponding to future quality enhancements of single cell genomics. (ZIP) [file pcbi.1004983.s002.zip › Clusters/Clusters_Param_20_20_pval_95_SNP7_Res_Future.jpg]

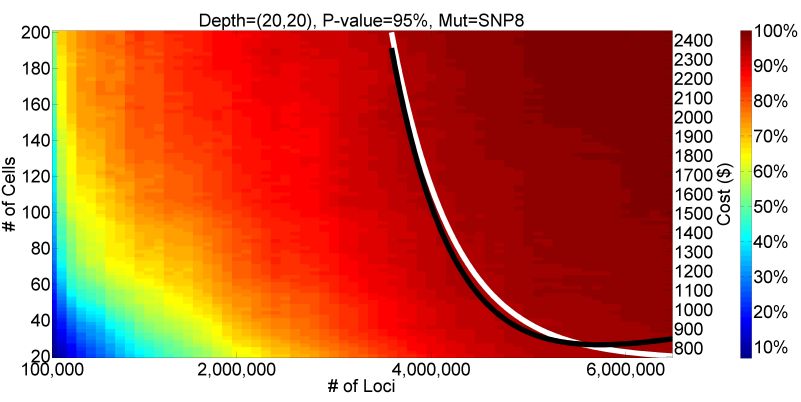

Supplement: S2 File — Results include figures similar to Figs 1B, 2B and 3B but using STR mutations with mutation rates 10−3 and 10−5 and SNV mutations with mutation rates 10−7, 10−8, 10−9 using current and improved values for ADO and noise corresponding to future quality enhancements of single cell genomics. (ZIP) [file pcbi.1004983.s002.zip › Clusters/Clusters_Param_20_20_pval_95_SNP8_Res.jpg]

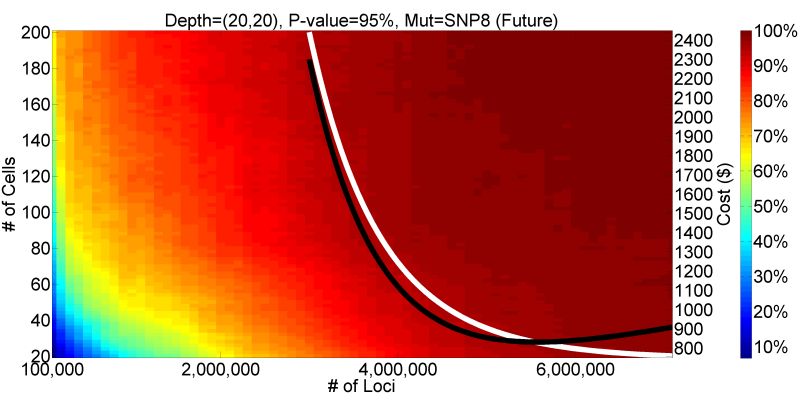

Supplement: S2 File — Results include figures similar to Figs 1B, 2B and 3B but using STR mutations with mutation rates 10−3 and 10−5 and SNV mutations with mutation rates 10−7, 10−8, 10−9 using current and improved values for ADO and noise corresponding to future quality enhancements of single cell genomics. (ZIP) [file pcbi.1004983.s002.zip › Clusters/Clusters_Param_20_20_pval_95_SNP8_Res_Future.jpg]

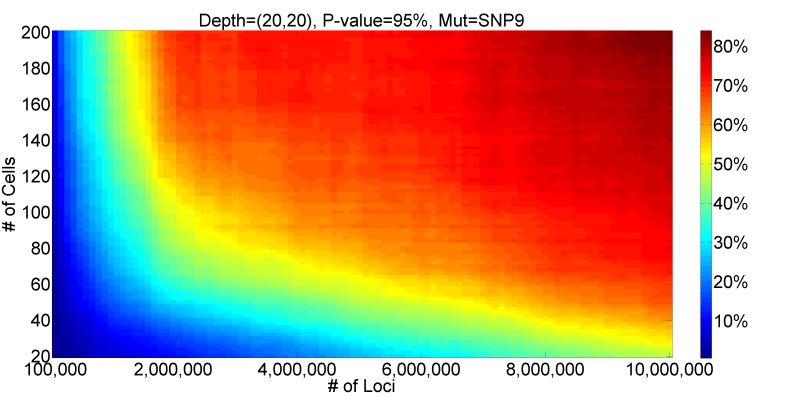

Supplement: S2 File — Results include figures similar to Figs 1B, 2B and 3B but using STR mutations with mutation rates 10−3 and 10−5 and SNV mutations with mutation rates 10−7, 10−8, 10−9 using current and improved values for ADO and noise corresponding to future quality enhancements of single cell genomics. (ZIP) [file pcbi.1004983.s002.zip › Clusters/Clusters_Param_20_20_pval_95_SNP9_Res.jpg]

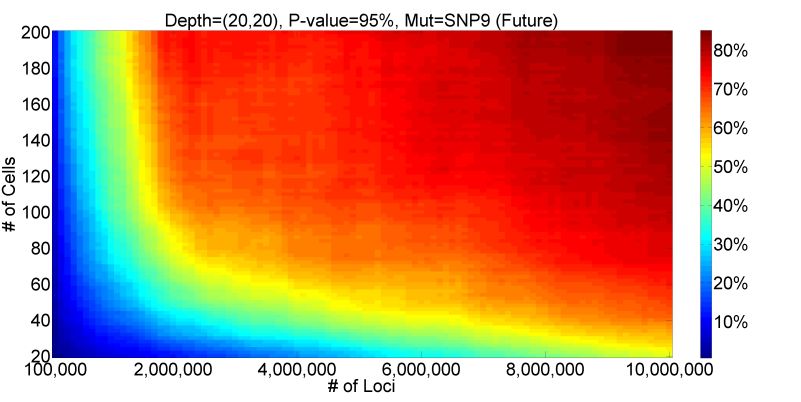

Supplement: S2 File — Results include figures similar to Figs 1B, 2B and 3B but using STR mutations with mutation rates 10−3 and 10−5 and SNV mutations with mutation rates 10−7, 10−8, 10−9 using current and improved values for ADO and noise corresponding to future quality enhancements of single cell genomics. (ZIP) [file pcbi.1004983.s002.zip › Clusters/Clusters_Param_20_20_pval_95_SNP9_Res_Future.jpg]

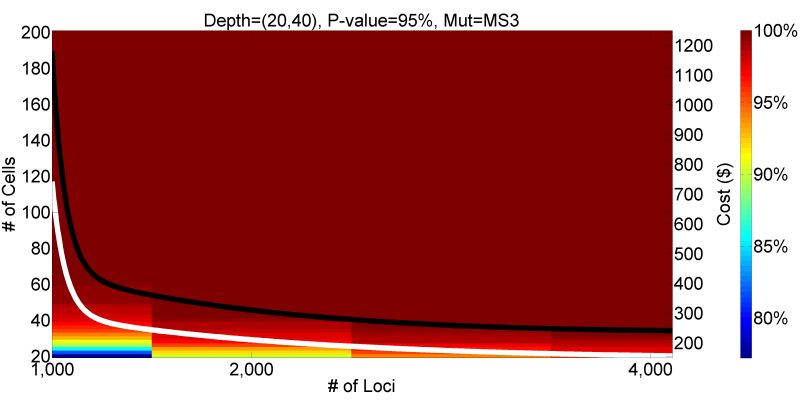

Supplement: S2 File — Results include figures similar to Figs 1B, 2B and 3B but using STR mutations with mutation rates 10−3 and 10−5 and SNV mutations with mutation rates 10−7, 10−8, 10−9 using current and improved values for ADO and noise corresponding to future quality enhancements of single cell genomics. (ZIP) [file pcbi.1004983.s002.zip › Clusters/Clusters_Param_20_40_pval_95_MS3_Res.jpg]

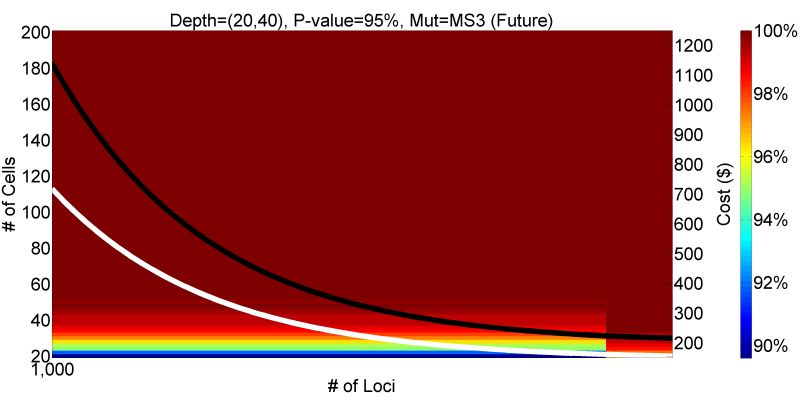

Supplement: S2 File — Results include figures similar to Figs 1B, 2B and 3B but using STR mutations with mutation rates 10−3 and 10−5 and SNV mutations with mutation rates 10−7, 10−8, 10−9 using current and improved values for ADO and noise corresponding to future quality enhancements of single cell genomics. (ZIP) [file pcbi.1004983.s002.zip › Clusters/Clusters_Param_20_40_pval_95_MS3_Res_Future.jpg]

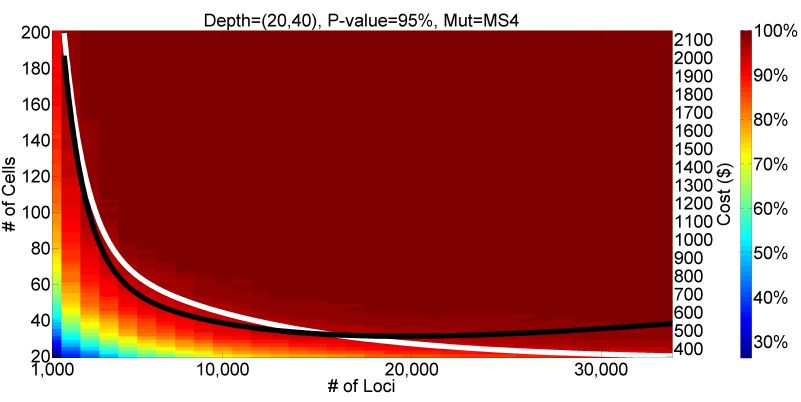

Supplement: S2 File — Results include figures similar to Figs 1B, 2B and 3B but using STR mutations with mutation rates 10−3 and 10−5 and SNV mutations with mutation rates 10−7, 10−8, 10−9 using current and improved values for ADO and noise corresponding to future quality enhancements of single cell genomics. (ZIP) [file pcbi.1004983.s002.zip › Clusters/Clusters_Param_20_40_pval_95_MS4_Res.jpg]

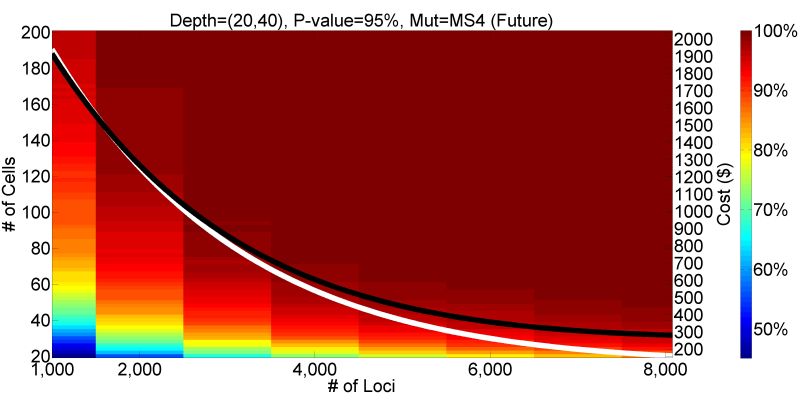

Supplement: S2 File — Results include figures similar to Figs 1B, 2B and 3B but using STR mutations with mutation rates 10−3 and 10−5 and SNV mutations with mutation rates 10−7, 10−8, 10−9 using current and improved values for ADO and noise corresponding to future quality enhancements of single cell genomics. (ZIP) [file pcbi.1004983.s002.zip › Clusters/Clusters_Param_20_40_pval_95_MS4_Res_Future.jpg]
