# Supplementary figures and images for: Accuracy of Answers to Cell Lineage Questions Depends on Single-Cell Genomics Data Quality and Quantity (part 3 of 3)
Source: PLoS Comput Biol. 2016 Jun 13;12(6):e1004983. doi: 10.1371/journal.pcbi.1004983 (PMC4905655; doi:10.1371/journal.pcbi.1004983)

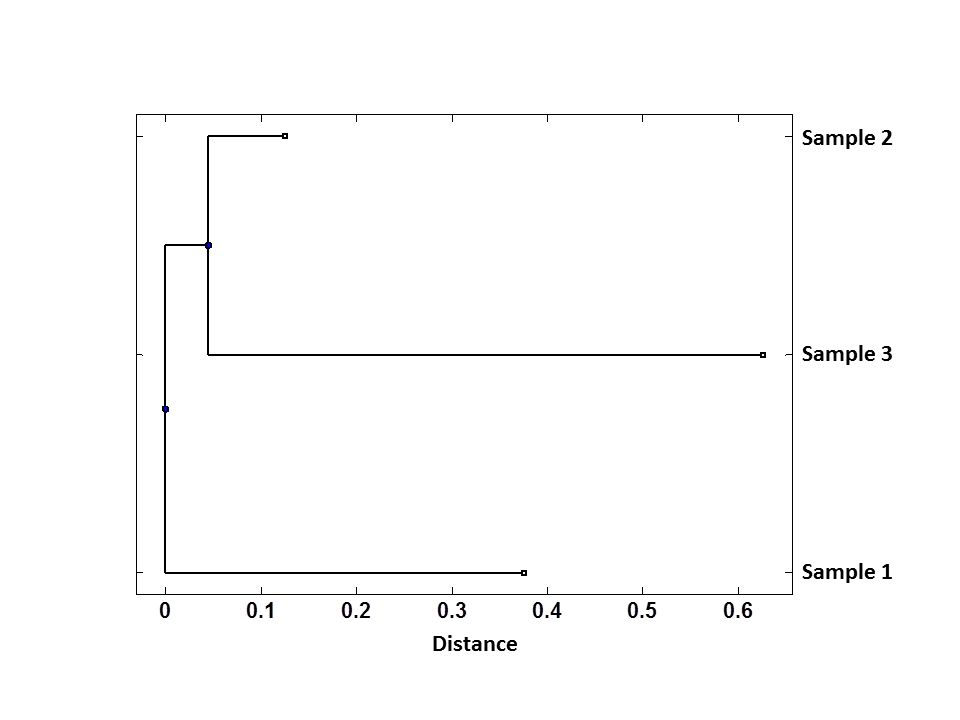

Supplement: S1 Fig — (TIF) [file pcbi.1004983.s003.tif]
